# Equations of State and Crystal Structures of KCaPO_4_, KSrPO_4_, and K_2_Ce(PO_4_)_2_ under High Pressure: Discovery of a New Polymorph of KCaPO_4_

**DOI:** 10.1021/acs.cgd.2c01547

**Published:** 2023-02-28

**Authors:** Daniel Errandonea, Srungarpu N. Achary, Daniel Diaz-Anichtchenko, Enrico Bandiello, Tomas Marqueño, Rakesh Shukla, Avesh K. Tyagi, Catalin Popescu, Frederico G. Alabarse

**Affiliations:** † Departamento de Física Aplicada-ICMUV, MALTA Consolider Team, Universidad de Valencia, Dr. Moliner 50, Burjassot, Valencia 46100, Spain; ‡ Bhabha Atomic Research Centre, Solid State Chemistry Section, Chemistry Division, Bhabha Atomic Research Centre, Trombay, Mumbai 400 085, India; § Instituto de Diseño para la Fabricación y Producción Automatizada, MALTA Consolider Team, Universitat Politècnica de Valéncia, Valencia 46022, Spain; ∥ CELLS—ALBA Synchtrotron Light Facility, Cerdanyola del Valles E-08290, Barcelona, Spain; ⊥ Elettra Sincrotrone Trieste, Trieste 34149, Italy

## Abstract

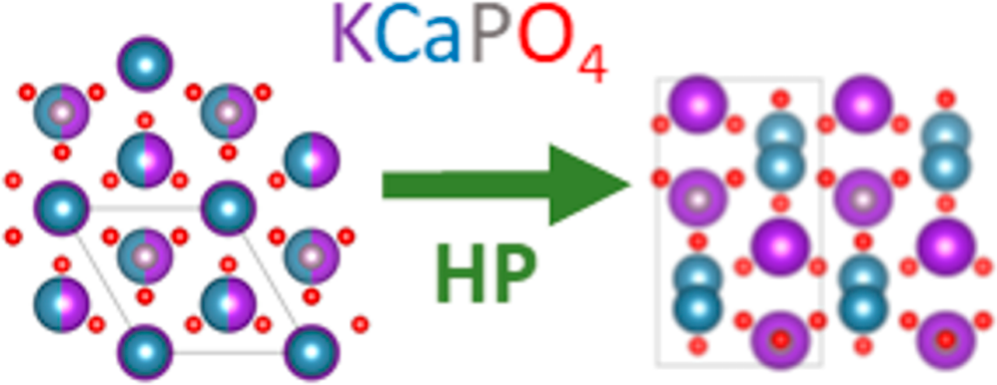

We have studied by means of angle-dispersive powder synchrotron X-ray diffraction the structural behavior of KCaPO_4_, SrKPO_4_, and K_2_Ce(PO_4_)_2_ under high pressure up to 26, 25, and 22 GPa, respectively. For KCaPO_4_, we have also accurately determined the crystal structure under ambient conditions, which differs from the structure previously reported. Arguments supporting our structural determination will be discussed. We have found that KCaPO_4_ undergoes a reversible phase transition. The onset of the transition is at 5.6 GPa. It involves a symmetry decrease. The low-pressure phase is described by space group *P*3̅*m*1 and the high-pressure phase by space group *Pnma*. For KSrPO_4_ and K_2_Ce(PO_4_)_2_, no evidence of phase transitions has been found up to the highest pressure covered by the experiments. For the three compounds, the linear compressibility for the different crystallographic axes and the pressure–volume equation of states are reported and compared with those of other phosphates. The three studied compounds are among the most compressible phosphates. The results of the study improve the knowledge about the high-pressure behavior of complex phosphates.

## Abstract 2

The present work focuses on the high-pressure behavior of complex orthophosphates; phase transitions and compressibilities are studied by X-ray diffraction.

## Introduction

Inorganic phosphates show a diversity of crystal structures because of their flexibility to accommodate cations with different oxidation states and ionic radii and linkages of phosphate groups, which, in turn, favor the formation of compounds with a wide variety of chemical compositions.^1^ The most common phosphates are orthophosphates based on the oxianion [PO_4_]^3–^, which forms complexes with almost all the cations in the periodic table. Orthophosphates are especially important because of their key roles in biochemistry, biogeochemistry, and ecology and their economic importance for agriculture and industry as lithium-ion technology in the age of electric vehicles.^2,3^ Besides, they often form water-insoluble or low-leaching complexes with lanthanides and actinides, which makes them promising matrices for separation or immobilization of heavy elements from environmental or nuclear waste.^4,5^ Also, orthophosphates have also been of interest for several technologically relevant applications, like efficient X-ray scintillation and display materials, and for lightning and stimulated emission sources due to their wide optical band gaps and efficient absorption in the ultraviolet region.^6−9^ In addition to their technological relevance, the crystal chemistry of orthophosphates has also drawn significant attention for decades due to their structural diversity arising from composition and external parameters such as pressure and temperature.^1,10,11^ In fact, these external thermodynamic variables are known to influence the structure of orthophosphates and lead to formation of denser polymorphs, which are invariably unstable under ambient conditions. Pressure is a well-established and versatile tool in materials research, as it can modify atomic interactions and chemical bonding, leading in many different oxides to the occurrence of pressure-driven phase transitions.^9−11^ Multiple high-pressure (HP) studies have been performed in APO_4_-type phosphates,^12−20^ where A is a trivalent cation. These APO_4_-type compounds crystallizing in the zircon-type or monazite-type structures usually undergo phase transitions only at pressure beyond 20 GPa.^12,13^ However, compounds with more open crystal structures, like berlinite-type AlPO_4_ and FePO_4_, undergo phase transitions at relatively lower pressures, 15 and 3 GPa, respectively.^14,15^ Similarly, in several orthophosphates with multiple cations, phase transitions have been reported at relatively low pressure. For instance, in BaZr(PO_4_)_2_, transitions happen at 0.4 and 8 GPa^16^ and in Pb_3_(PO_4_)_2_ at 1.7 GPa.^17^ In contrast, K_2_Ce(PO_4_)_2_ undergoes a phase transition at 8.6 GPa,^18^ and in Ca_3_(PO_4_)_2_ and Sr_3_(PO_4_)_2_, no evidence of phase transitions was reported in studies performed up to 20 GPa.^19,20^ Clearly, the systematic behavior of orthophosphates under HP is extremely complex and is yet to derive any systematic trend, and hence, additional studies are needed to fully understand it.

In this work, we have investigated the HP behavior of three orthophosphates KCaPO_4_, KSrPO_4_, and K_2_Ce(PO_4_)_2_ by means of *in situ* HP powder X-ray diffraction (XRD). KCaPO_4_ and KSrPO_4_ are two of the stable phases in the K_2_O-Ca/SrO-P_2_O_5_ system and have been investigated for their physical properties which make them suitable for solid state lighting, white light-emitting diode applications, or radiation dosimetry.^21−23^ Although the composition and properties of KCaPO_4_ and KSrPO_4_ have been delineated since long, no studies on their HP behavior are available in the literature till date. The crystal structure of KCaPO_4_ was initially assigned to a trigonal or pseudohexagonal lattice in analogy to α-or β-K_2_SO_4_ structures.^24^ For KSrPO_4_, an orthorhombic lattice closely related to β-K_2_SO_4_ has been assigned.^24−26^ However, the discrepancy for the model crystal structure of KCaPO_4_ remained due to an admixture of high- and low-temperature phases. K_2_Ce(PO_4_)_2_ is a recently reported complex phosphate with uncommon Ce^4+^ ions.^27^ Owing to the stabilized Ce^4+^ ions, it shows catalytic properties for organic synthesis involving redox reactions,^28^ while the zeolitic-like structure makes it a promising material for selective separation of radioactive ions from dilute solutions.^29^ Temperature-dependent structural studies on K_2_Ce(PO_4_)_2_ indicated a first-order reconstructive monoclinic (space group *P*2_1_/*n*) to tetragonal (space group *I*4_1_/*amd*) structural transition at high temperature, while no transitions were observed at low temperature.^30,31^ The structure and stability of K_2_Ce(PO_4_)_2_ suggest that it can be a promising X-ray scintillator and luminescent material.^27,32^ Preliminarily, HP Raman spectroscopic investigation suggested a possible structural transition in K_2_Ce(PO_4_)_2_ at 8.6 GPa.^18^ A change in the slopes of pressure-dependent mode frequency or the appearance of several new Raman modes is observed at the transition. However, the crystal structure of the HP phase remained undetermined in the study. In this work, we explore the structural stability of the aforementioned compounds, and additionally, we determine their compressibility and pressure–volume equation of state (EOS). Furthermore, the crystal structure of KCaPO_4_ under ambient conditions has been reevaluated. Our results are compared with those of previous studies on analogous phosphates.

## Methods

KCaPO_4_ and KSrPO_4_ were synthesized via a solid-state method using stoichiometric amounts of CaCO_3_ and SrCO_3_ (99.0%, Alfa Aesar) and KH_2_PO_4_ (99.0%, Loba Chemicals). A homogeneous mixture of the reactants, in the powder form, was placed in a platinum crucible and heated on a hot plate for 30 min at 450 °C. For both samples, the resulting products were homogenized and pelletized and heated again at 500 °C (12 h), 700 °C (18 h), 800 °C (18 h), and then at 850 °C (12 h) using a platinum crucible. After each treatment, the products were reground and pelletized, while the progress of the reaction was monitored by powder XRD after each heat treatment. Formation of single-phase materials was observed in both phosphates after this repeated heat treatment. The obtained KCaPO_4_ and KSrPO_4_ products were finally reground and pressed into pellets and heated again at 900 °C for 12 h. This product was used for our HP studies. K_2_Ce(PO_4_)_2_ was prepared by a solid state reaction of CeO_2_ and freshly prepared KPO_3_. KPO_3_ was prepared by decomposing KH_2_PO_4_ on a hot plate at 450 °C. The glass-like transparent KPO_3_ sample was finely ground and thoroughly mixed with CeO_2_ (99.5%, Indian Rare Earth Ltd.) in a 2:1 molar ratio and pelletized. The pellets were initially heated at 500 °C for 12 h and then at 750 °C for 12 h using a platinum crucible. The final product was characterized by powder XRD and used for HP studies.

HP angle-dispersive powder XRD experiments were carried out at the BL04-MSPD beamline in the ALBA-CELLS synchrotron^33^ and at the Xpress beamline at the Elettra Synchrotron Facility.^34^ Membrane-type diamond-anvil cells were used to apply pressure. The diameters of the culets of the diamonds were 350–500 μm. Stainless-steel 301 was used as the gasket material. The gasket was pre-indented to a thickness of 40 μm and a 150–200 μm hole. A 16:3:1 mixture of methanol, ethanol, and water was used as the pressure-transmitting medium. In the experiment performed in KCaPO_4_, we used the EOS of Cu to determine pressure.^35^ In the other two experiments, we used the ruby scale to measure pressure.^36^ In the three experiments, pressures were determined with an accuracy better than 0.05 GPa. KCaPO_4_ and KSrPO_4_ were measured at ALBA using a monochromatic X-ray beam with a wavelength of 0.4246 and 0.4642 Å, respectively. The size of the beam spot was 20 × 20 μm. Diffraction images were recorded using a Rayonix SX165 charge-coupled device. The detector parameters were calibrated using LaB_6_ as the standard. K_2_Ce(PO_4_)_2_ was measured at Elettra using a monochromatic X-ray beam with a wavelength of 0.4956 Å. The size of the beam spot was 80 μm in diameter. Diffraction images were recorded using a PILATUS 3S 6M detector. The detector parameters were calibrated using CeO_2_ as the standard. The diffraction images were integrated into intensity vs 2θ XRD patterns using the DIOPTAS program.^37^ Rietveld refinements and Le Bail fits were performed using the FullProf suite.^38^

## Results and Discussion

### Crystal Structure of KCaPO_4_

As mentioned in the Introduction, the crystal structure of KCaPO_4_ has been first reported by Bredig in analogy to K_2_SO_4_ structures, and a trigonal lattice with unit-cell parameters *a* = 5.58 Å and *c* = 7.60 Å (space group *P*3̅*m*1) has been assigned.^23^ In addition, the structure has been proposed by considering a statistical distribution of Ca^2+^ and K^+^ ions. Since the number of electrons of Ca^2+^ (20) and K^+^ (19) cations differs by only one, up to now, it has been assumed that K and Ca occupy the same sites randomly with a partial occupation of 0.5. The atomic coordinates as included in the Inorganic Crystal Structure Database (ICSD), collection code 27958, are given in Table 1. In all the following studies, the crystal structure reported by Bredig^24^ has been used to identify the KCaPO_4_ phase. However, the atomic coordinates were not accurate enough to reveal the structural arrangements of the atoms in the unit cell.

**Table 1:** Atomic Positions and Occupations (Occ.) in the Crystal Structure of KCaPO_4_ Reported in the ICSD, Structure Number 27958

atom	site	*x*	*Y*	*Z*	Occ.
O_1_	2d	2/3	1/3	0.460	1
O_2_	6i	0.200	–0.200	0.200	1
Ca_1_	1a	0	0	0	0.5
K_1_	1a	0	0	0	0.5
Ca_2_	1b	0	0	1/2	0.5
K_2_	1b	0	0	1/2	0.5
Ca_3_	2d	2/3	1/3	0.875	0.5
K_3_	2d	2/3	1/3	0.875	0.5
P	2d	2/3	1/3	0.270	1

**Figure 1:**
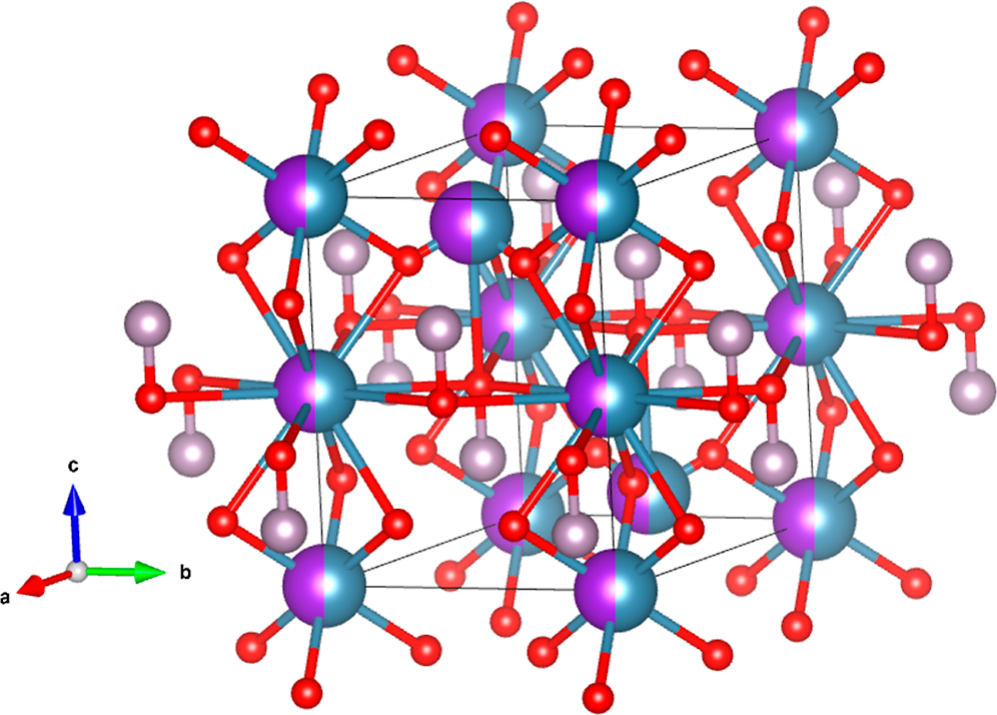
Schematic view of the KCaPO_4_ crystal structure reported by Bredig.^24^ Calcium/potassium shading atoms are represented in blue/purple with 50% of occupancy. Phosphor atoms are shown in the gray color and oxygen atoms in the red color. In the figure, the unusual coordination of phosphor atoms can be seen.

The structure as reported by Bredig^24^ is represented in Figure 1. This structure has several unusual features which suggest that it is probably not the correct crystal structure. The first one is the coordination of phosphor, which is only coordinated with one oxygen atom (P–O distance 1.44 Å), while other P–O distances are 2.85 Å. This arrangement is quite unusual for phosphates, in which the PO_4_^3–^ anion is generally a regular or nearly regular tetrahedron. Indeed, such PO_4_^3–^ is expected for K^+^Ca^2+^PO_4_^3–^ also. Another unexpected feature is that Ca and K could occupy randomly with equal probability the 1a site (corners of the structure) and the 1b site (center of the edges and then ran parallel to the *c*-axis). Notice that both sites have a very different packing density. Atoms in 1a are octahedrally coordinated with oxygen atoms with a bond distance of 2.459 Å, which is usually expected for Ca–O bonds. Atoms in 1b have a different coordination environment, being coordinated with six oxygen atoms at 2.898 Å and six equatorial oxygen atoms at 3.236 Å. Thus, it is more reasonable to expect that Ca, which has a much smaller ionic radius than K, would occupy the 1a site, and K would occupy the 1b site. On top of this, cations at 2b (Ca or K) are only linked to three oxygen atoms with a bond distance of 1.409 Å, which is also very odd. Additionally, Louer *et al.*(39) reported the crystal structure of a hydrated phase, KCaPO_4_·H_2_O, on a monoclinic lattice from powder XRD data. The structure is explained, as expected, by regular PO_4_ tetrahedra where the Ca and K are completely ordered, maintaining eight coordinated polyhedral units around them. Furthermore, the Ca atoms are connected to six oxygen atoms of four PO_4_ tetrahedra and additional two oxygen atoms from water molecules, while the K atoms are linked to eight oxygen atoms of four tetrahedral PO_4_ units. Thus, the coordination of K and Ca reported for KCaPO_4_ by Bredig^24^ needs rectification, and the described drawbacks might be due to poor data quality or refinements. Similar difference is also observed in comparison to the structure of trigonal NaBaPO_4_ reported by Launay *et al.*(25) To clarify these issues, we have measured a powder XRD pattern of KCaPO_4_ under ambient conditions and refined the crystal structure, which turns out to be different than the previously reported structure.

**Figure 2:**
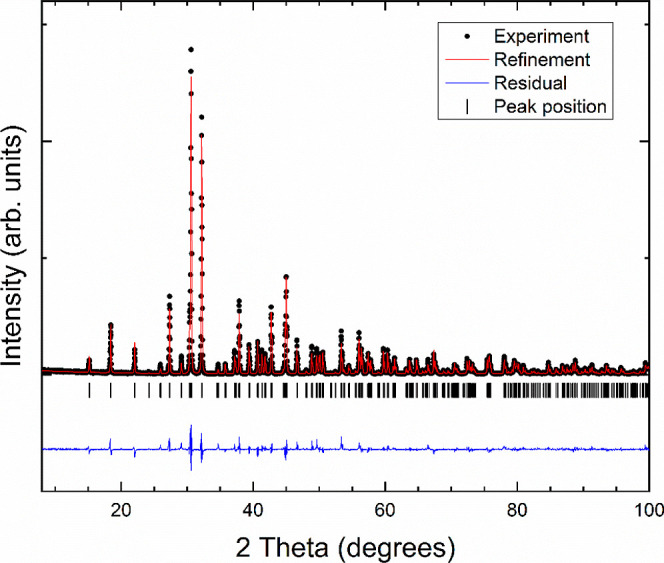
XRD pattern measured in KCaPO_4_ under ambient conditions (λ = 0.4246 Å).

For the structural assignation, we first indexed the XRD patterns using DICVOL^40^ and then assigned the symmetry. It is found that the space group *P*3̅*m*1 gives the best figure of merit, which agrees with the space group reported by Bredig.^24^ Subsequently, we determine atomics positions from Rietveld refinements, which were performed using as the starting point the known crystal structure but under the assumption that Ca atoms occupy the 1a site and K atoms occupy the 1b site, both with full occupation. In addition, they also share a 2d position as in the previously proposed crystal structure.^24^ Besides, the oxygen (O_2_) positions are assigned by rotating them by 120° in the ab-plane. Figure 2 shows the Rietveld refinement pattern of XRD data of KCaPO_4_. The structure that we have obtained gives better goodness-of-fit parameters than the structure currently used in the literature,^23^ and it is more reasonable from a crystal chemistry point of view. The goodness-of-fit parameters are w*R*_p_ = 2.40%, *R*_p_ = 1.85%, *R*(*F*^2^) = 7.44%, and χ^2^ = 1.37. If the structure reported by Bredig^24^ was assumed, the goodness-of-fit parameters were w*R*_p_ = 21.10%, *R*_p_ = 15.76%, *R*(*F*^2^) = 26.64%, and χ^2^ = 2.54. The improvement in the refinement and the fact that our reported structure is chemically more plausible support the crystal structure that we are proposing. The obtained unit-cell parameters are *a* = 5.4994(5) Å and *c* = 7.5701(7) Å. The atomic positions are reported in Table 2. A complete crystallographic information of the structures can be obtained from the Cambridge Crystallographic Data Centre (CCDC) under deposition number 2192664.

**Table 2:** Atomic Positions and Occupations (Occ.) of the Crystal Structure of KCaPO_4_ Here Determined

atom	site	*x*	*y*	*z*	Occ.
O_1_	2d	2/3	1/3	0.4750(9)	1
O_2_	6i	–0.1948(9)	0.1948(9)	0.2311(9)	1
Ca_1_	1a	0	0	0	1
K_1_	1b	0	0	1/2	1
Ca_2_	2d	2/3	1/3	0.7366(2)	0.5
K_2_	2d	2/3	1/3	0.7366(2)	0.5
P	2d	2/3	1/3	0.2910(1)	1

**Figure 3:**
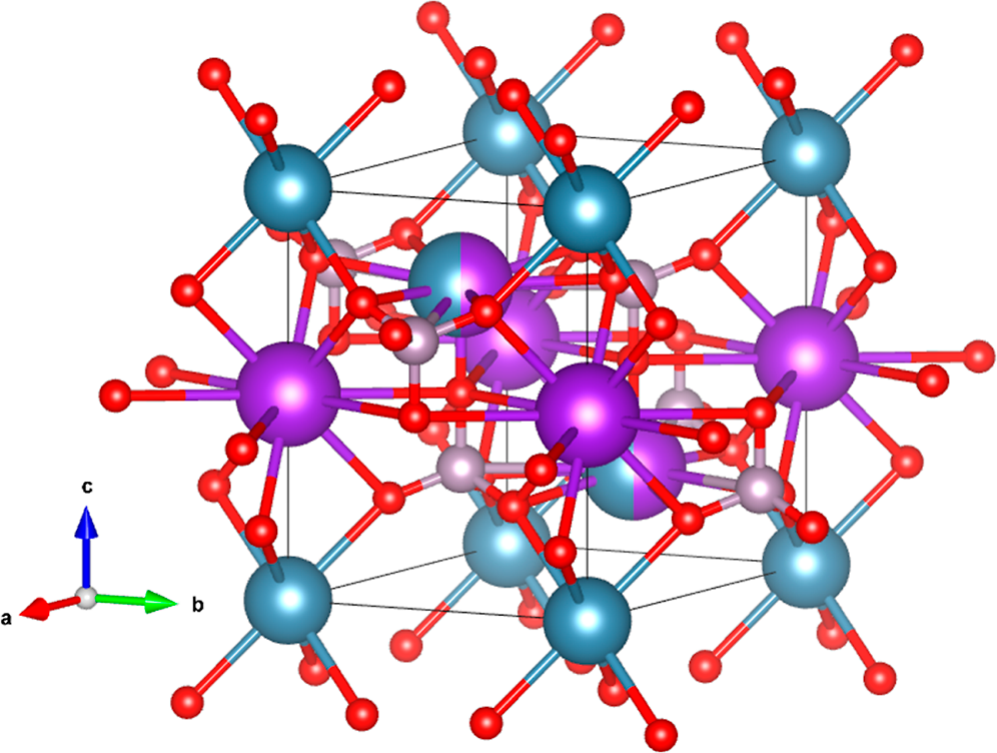
Schematic view of the crystal structure of KCaPO_4_ proposed here. Calcium atoms are represented in blue, while potassium atoms are represented in purple (shading atoms represent 50% of occupancy). Phosphor atoms are in gray, and oxygen atoms are in the red color. It can be seen that in this structure, phosphor is in tetrahedral coordination.

The proposed crystal structure is represented in Figure 3. It has two formula units per unit cell and is isomorphic to the structure of NaBaPO_4_.^25^ It contains chains aligned along the *c*-axis and composed of alternate CaO_6_ octahedra and KO_12_ dodecahedra that share triangular faces. The distorted CaO_6_ units share oxygen atoms with six separate PO_4_ tetrahedra. Each 12-fold coordination polyhedron of K is formed by 6 oxygen atoms in a similar configuration to that in the CaO_6_ octahedron, while additional 6 oxygen atoms are nearly coplanar and lie approximately in the equatorial plane of this cation. The remaining cations of Ca and K atoms are randomly located with a 1/2 occupation at sites of 3*m* symmetry, being aligned along the *c*-axis with P atoms. These Ca(K) cations are coordinated with seven oxygen atoms forming a hexagonal pyramid. The cations are at the center of the base of the pyramid connected to six nearly coplanar oxygen atoms, with bond distance 2.773(2) Å. The seventh oxygen atom is in the vertex of the pyramid. This bond is shorter than the other six bonds [bond distance 1.980(1) Å], and the oxygen atom is shared with a PO_4_ tetrahedron. On the opposite side of the vertex, there are three oxygen atoms at 3.770(2) Å from Ca(K), making a Ca(K)O_10_ pseudo-polyhedron. The bond distances are 2.550(2) Å in the CaO_6_ octahedron, 2.754(2) and 3.180(3) Å in the KO_12_ polyhedron, and 1.393(1) and 1.395(1) Å in the PO_4_ tetrahedron which is nearly regular. PO_4_ and Ca(K)O_10_ make a linear chain. These chains are the link between CaO_6_–KO_12_ units, giving three-dimensional cohesion to the crystal structure.

### KCaPO_4_ under High Pressure

**Figure 4:**
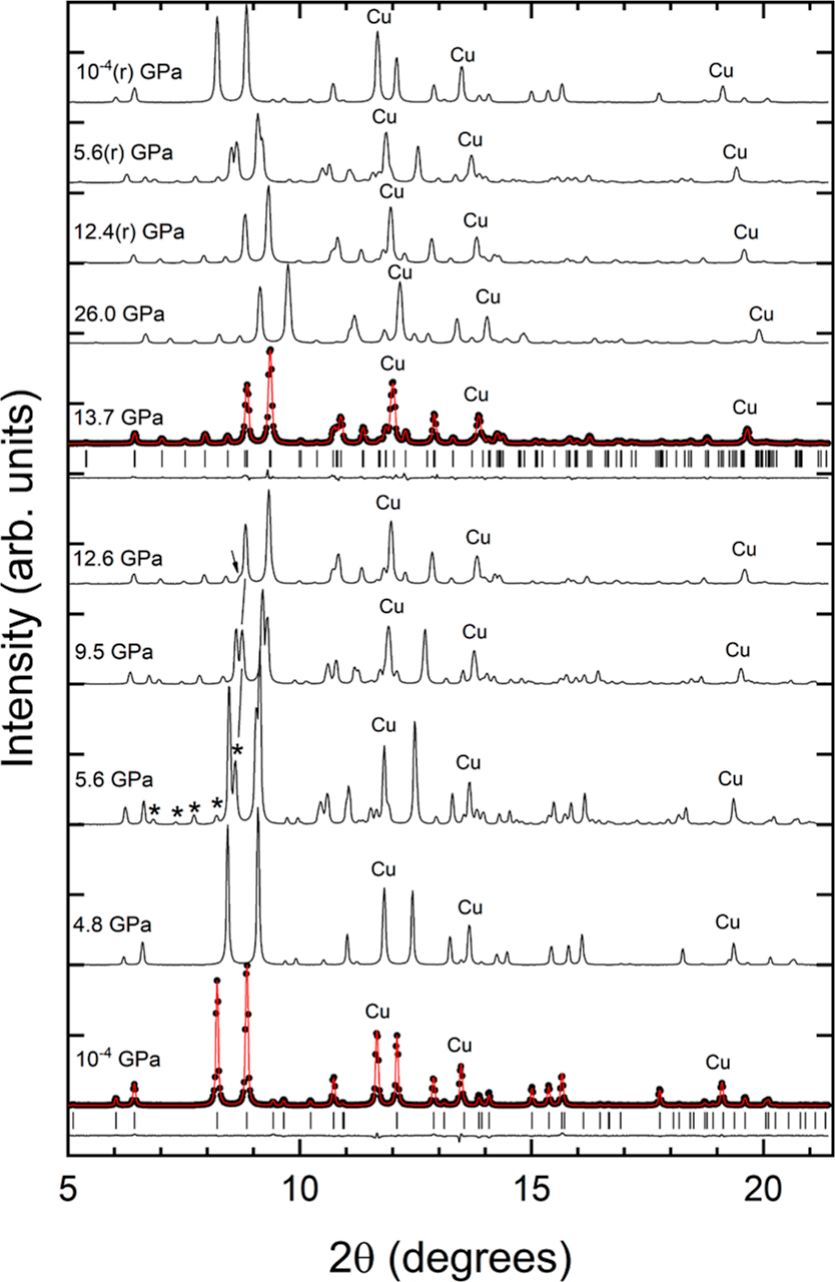
HP XRD patterns measured for KCaPO_4_ (λ = 0.4246 Å). At 10^–4^ and 13.7 GPa, experiments are shown as solid symbols, refinements as red lines, and residuals as black lines. The ticks show the position of calculated reflections. Pressures are indicated in the figure; (r) is used to identify experiments performed during pressure release. Cu peaks used for pressure determination are labeled as Cu. At 5.6 GPa, the asterisk shows emerging peaks. At 12.6 GPa, the arrows show a weak peak from the LP phase. The peak at 5.6, 9.5, and 12.6 GPa connected by solid lines is the peak discussed in the text to show the gradual transformation of the sample.

In Figure 4, we present a selection of XRD patterns collected under compression up to 26 GPa. From ambient pressure (10^–4^ GPa) up to 4.8 GPa, the XRD patterns can be undoubtedly assigned to the low-pressure (LP) phase. In addition to the peaks from the sample, three peaks from Cu (the pressure standard) are detected. Evidence of the correct structural identification can be seen in the Rietveld refinement of the experiment carried out at 10^–4^ GPa (see Figure 4). When increasing the pressure to 5.6 GPa, several extra peaks appear in the XRD pattern. They can be clearly identified in the low-angle part of the XRD pattern and are denoted with asterisks in Figure 4. We consider this fact an indication of the onset of a structural phase transition. As pressure increases, the extra peaks become stronger, and the peaks of the LP phase become weaker. To highlight this phenomenon, in Figure 4, we follow with a solid line the pressure evolution of one peak of the emerging phase, which is at 2θ = 8.6° at 5.6 GPa. As this peak grows, the peak from the LP phase on its left shrinks even if it can be detected up to 12.6 GPa as a shoulder to the more intense peak (as indicated by an arrow). We have been able to identify all peaks at 13.7 GPa as due to Cu and a phase different than the LP structure. Thus, we assume that the phase transition is completed at 13.7 GPa. The phase coexistence from 5.6 to 12.6 GPa suggests that the sample undergoes a first-order phase transition.

The XRD pattern measured at 13.7 GPa was indexed using DICVOL^40^ to determine the unit-cell parameters. From a list of the first 10 peaks (to avoid overlap with Cu peaks), the highest figures of merit have been obtained for an orthorhombic lattice with unit-cell parameters *a* = 6.930(1) Å, *b* = 5.196(1) Å, and *c* = 9.034(2) Å. These parameters resemble those of the orthorhombic structures of KSrPO_4_ [*a* = 7.352(2) Å, *b* = 5.561(1) Å, and *c* = 9.642(3) Å]^26^ and KBaPO_4_ [*a* = 7.709(4) Å, *b* = 5.663(4) Å, and *c* = 9.972(5) Å].^41^ Both compounds are isomorphic and described by the orthorhombic space group *Pnma*. According to the systematic approach proposed by Bastide, the pressure-induced transitions may occur from the structure of a given compound to that of a larger-cation-hosting compound.^42,43^ The presently observed unit cell is in accordance with the Bastide structural systematic approach. Thus, an isomorphic structure like KSrPO_4_ or KBaPO_4_ may be logical to consider model candidates for the HP phase of KCaPO_4_. A model using the unit-cell parameters from our XRD pattern observed at 13.7 GPa and atomic positions from KSrPO_4_ (substituting Sr for Ca) has allowed us to satisfactorily explain the complete XRD pattern. The quality of the refinement and the small residuals can be seen in Figure 4. The goodness-of-fit parameters are w*R*_p_ = 3.22%, *R*_p_ = 2.85%, *R*(*F*^2^) = 9.27%, and χ^2^ = 1.93. Thus, we consider that a very probable structure for the HP phase of KCaPO_4_ could be obtained. The atomic positions at 13.7 GPa are given in Table 3. The complete crystallographic data can be obtained from CCDC 2192910.

**Table 3:** Atomic Positions and Occupations (Occ.) of the HP Crystal Structure of KCaPO_4_ at 13.7 GPa

atom	site	*x*	*y*	*z*	Occ.
O_1_	4c	0.3145(9)	1/4	0.0663(9)	1
O_2_	8d	0.2921(9)	0.0252(9)	0.8447(9)	1
O_3_	4c	0.5095(9)	1/4	0.5883(9)	1
Ca	4c	0.9976(6)	1/4	0.1969(6)	1
K	4c	0.1590(6)	1/4	0.5858(6)	1
P	4c	0.2297(3)	1/4	0.9183(3)	1

**Figure 5:**
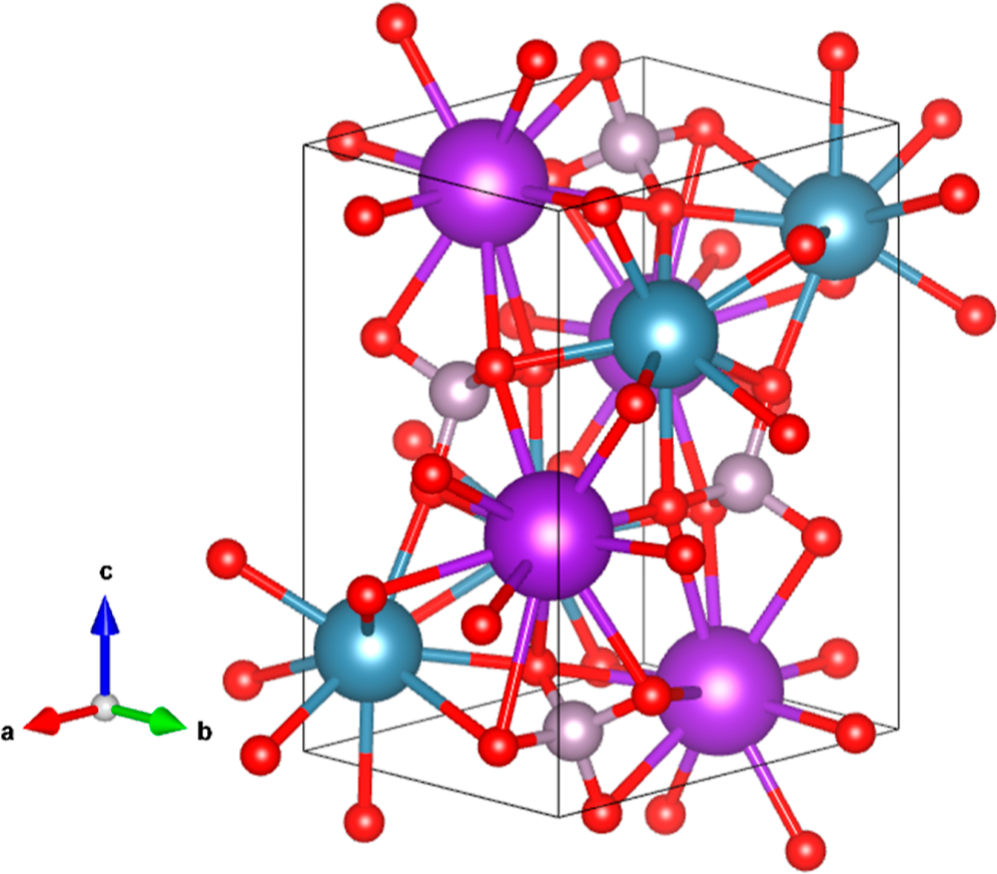
Schematic view of the HP crystal structure of KCaPO_4_. Calcium atoms are represented in blue. Potassium atoms are represented in purple, phosphor atoms in gray, and oxygen atoms in the red color.

The crystal structure of the HP phase is shown in Figure 5. It contains four formula units per unit cell, with no fractional occupations. Interestingly, *c/b* ≈√3 which implies that the crystal structure is ortho-hexagonal. Upon comparing the HP structure with the trigonal LP structure, it can be noticed that the lattice parameter *a* in the orthorhombic structure is nearly equal to the lattice parameter *c* in the trigonal structure. On the other hand, *b* and *c*/ of the orthorhombic structure are similar to the lattice parameter *a* in the trigonal structure. Thus, the structures of the transformed HP and LP phases of KCaPO_4_ are closely related, and they can be compared by considering the unit cell relations, as *a*_HP_ ∼ *c*_LP_, *b*_HP_ ∼ *a*_LP_, and .

**Figure 6:**
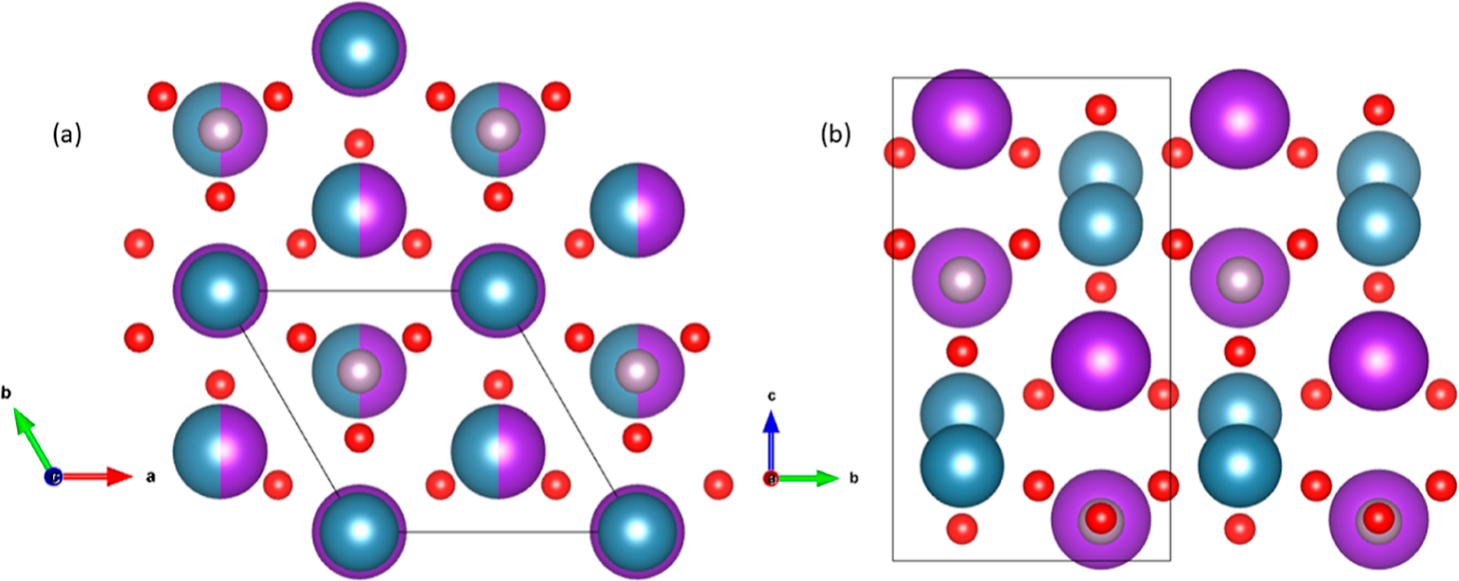
Perspectives of the LP (a) and HP (b) structures of KCaPO_4_. Calcium atoms are represented in blue. Potassium atoms are represented in purple, phosphor atoms in gray, and oxygen atoms in the red color.

The LP and HP structures of KCaPO_4_ are compared in Figure 6 where we present a perspective of each structure which highlights the similitudes and differences between them. The figure also allows us to understand the mechanism behind the transition. At the transition, there is not only a change of the axial ratio (*c/a* in the trigonal LP phase is 1.37, and *a/b* in the orthorhombic HP structure is 1.32) but also important atomic displacements which involve the formation of new bonds. Also, both structures are closely related to the LT and HT polymorphs of K_2_SO_4_.^44,45^ Essentially, both the structures are formed by packing of the PO_4_^3–^ anions and K^+^ and Ca^2+^ ions. This can be visualized easily from the cation-ordered HP KCaPO_4_. There is a stacking of K^+^ and PO_4_^3–^ layers sandwiched between layers of Ca^2+^ ions. This can be also seen in Figure 6. Calcium and potassium atoms are surrounded by PO_4_ tetrahedra in a compact arrangement. The PO_4_ tetrahedron is slightly distorted with an average P–O distance of 1.452(1) Å. The movements of the ions in the LP KCaPO_4_ under pressure resulted in a distorted structure which, in turn, resulted in an ortho-hexagonal arrangement. This also justifies our observation of the cation-ordered structure for LP KCaPO_4_.

Furthermore, in the HP phase, Ca is coordinated with 9 oxygen atoms, with an average bond distance of 2.553(5) Å, and K is coordinated with 11 oxygen atoms, with an average distance of 2.732(5) Å. The movement of ions in the *ab* plane of the trigonal structure, which is equivalent to the *bc*-plane of the orthorhombic phase, is mostly brought about by the compression of the *c*-axis of the trigonal phase. This also results in a better packing by rendering the effective coordination for the K and Ca atoms. This is also in accordance with the reconstructive nature of the LP to HP transformation. The observed coexistence of phases and the absence of a group–subgroup relationship between the symmetries of the two phases support the fact that the phase transition is of the first order. In addition, it may be mentioned here that it is also possible that the symmetry of the LP phase is further lower where the K_2_ and Ca_2_ atoms are orderly occupied in two distinct sites with half occupancy. Additional support to this conclusion will come when discussing the pressure dependence of the unit-cell volume.

Upon further compression beyond 13.7 GPa, we did not find any evidence of additional phase transitions. All XRD patterns measured up to 26 GPa can be identified within the same HP phase of the XRD pattern measured at 13.7 GPa. Figure 4 shows that the only changes from the XRD pattern at 26 GPa and the one at 13.7 GPa are the shift toward higher angles of peaks due to the contraction of unit-cell parameters, when pressure is increased. Pressure has been decreased from 26 GPa to ambient pressure (10^–4^ GPa) in three steps. At 12.4 GPa, only the HP phase has been observed. At 5.6 GPa, the coexistence of the two phases was observed, and at ambient pressure, the LP phase had been recovered, with the phase transition therefore being reversible.

**Figure 7:**
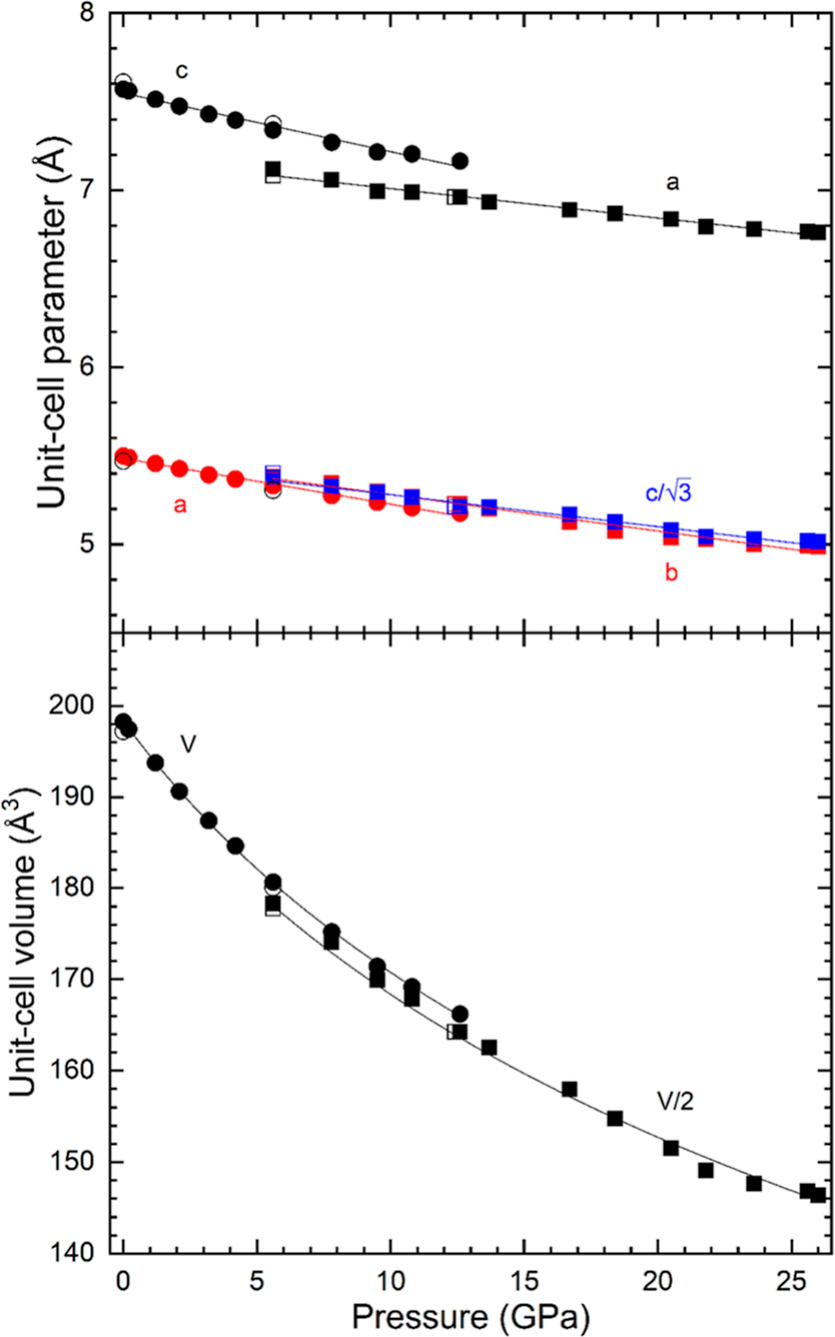
Pressure dependence of unit-cell parameters (top) and volume (bottom). Circles (squares) are the results from the low (high)-pressure phase. Solid (empty) data have been measured during compression (decompression). For the HP phase, we plot *c*/√3 and *V*/2 to facilitate comparison with the LP phase. Solid lines are the fits described in the text. Error bars are smaller than symbol size.

From measured XRD patterns, we have obtained the pressure dependence of unit-cell parameters for the LP and HP phases. The results are present in Figure 7, which shows that the unit-cell parameters follow a nearly linear dependence on pressure. As shown in the figure, at the phase transition, there is a discontinuity of the *c*-axis of the LP phase (which corresponds to the *a*-axis of the HP phase). There is also a 1.5% contraction of the unit-cell volume, which is larger than the error for volume determination. This supports the fact that the observed transition is of the first order. From the results, the linear compressibility has been determined for different axes (linear fits are shown as solid lines). In the LP phase, κ_a_ = 4.38(4) 10^–3^ GPa^–1^ and κ_c_ = 4.71(4) 10^–3^ GPa^–1^. Consequently, compression is slightly anisotropic, with the *c*-axis being the most compressible axis. In addition, for the HP, we have obtained κ_a_ = 2.31(8) 10^–3^ GPa^–1^, κ_b_ = 3.75(8) 10^–3^ GPa^–1^, and κ_c_ = 3.30(8) 10^–3^ GPa^–1^. Then, the HP phase has a highly anisotropic response to pressure, with the *a*-axis being the least compressible axis. As the *a*-axis of the HP phase is equivalent to the *c*-axis, the movement of ions might be restricted by the arrangement of ions to attain effective coordination for all. Regarding the pressure dependence of the unit-cell volume of the different structures, we have analyzed it using a third-order Birch–Murnaghan EOS.^46^ Fits have been carried out using EosFit7.^47^ The determined values for the ambient-pressure volume (*V*_0_), ambient-pressure bulk modulus (*K*_0_), and its pressure derivative (*K*_0_′) and the implied value of the second pressure derivative (*K*_0_″) are for the LP phase, *V*_0_ = 198.3(2) Å^3^, *K*_0_ = 49(2) GPa, *K*_0_′ = 4.03(0.28), and *K*_0_″ = −0.0796 GPa^–1^ and for the HP phase, *V*_0_ = 390.2(5) Å^3^, *K*_0_ = 50(2) GPa, *K*_0_′ = 3.97(0.33), and *K*_0_″ = −0.0769 GPa^–1^, respectively. The values obtained for *K*_0_′ in both fits indicate that the EOSs are compatible with a second-order EOS (*B*_0_′ = 4). The bulk moduli of both phases agree with each other within error bars. Since the values of *K*_0_ and *K*_0_′ from both phases agree within experimental uncertainties, both phases have a very similar volumetric resistance to compression.

### KSrPO_4_ under High Pressure

**Figure 8:**
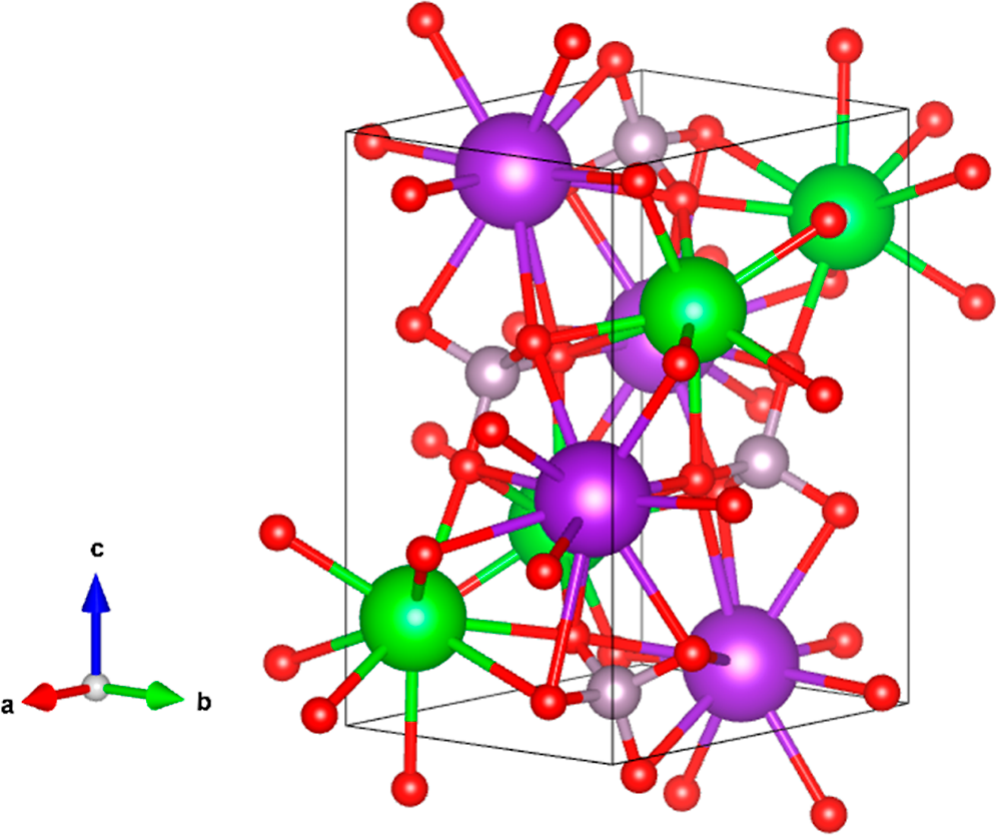
Schematic view of the crystal structure of KSrPO_4_. Strontium atoms are represented in green, potassium atoms in purple, phosphor atoms in gray, and oxygen atoms in the red color.

**Figure 9:**
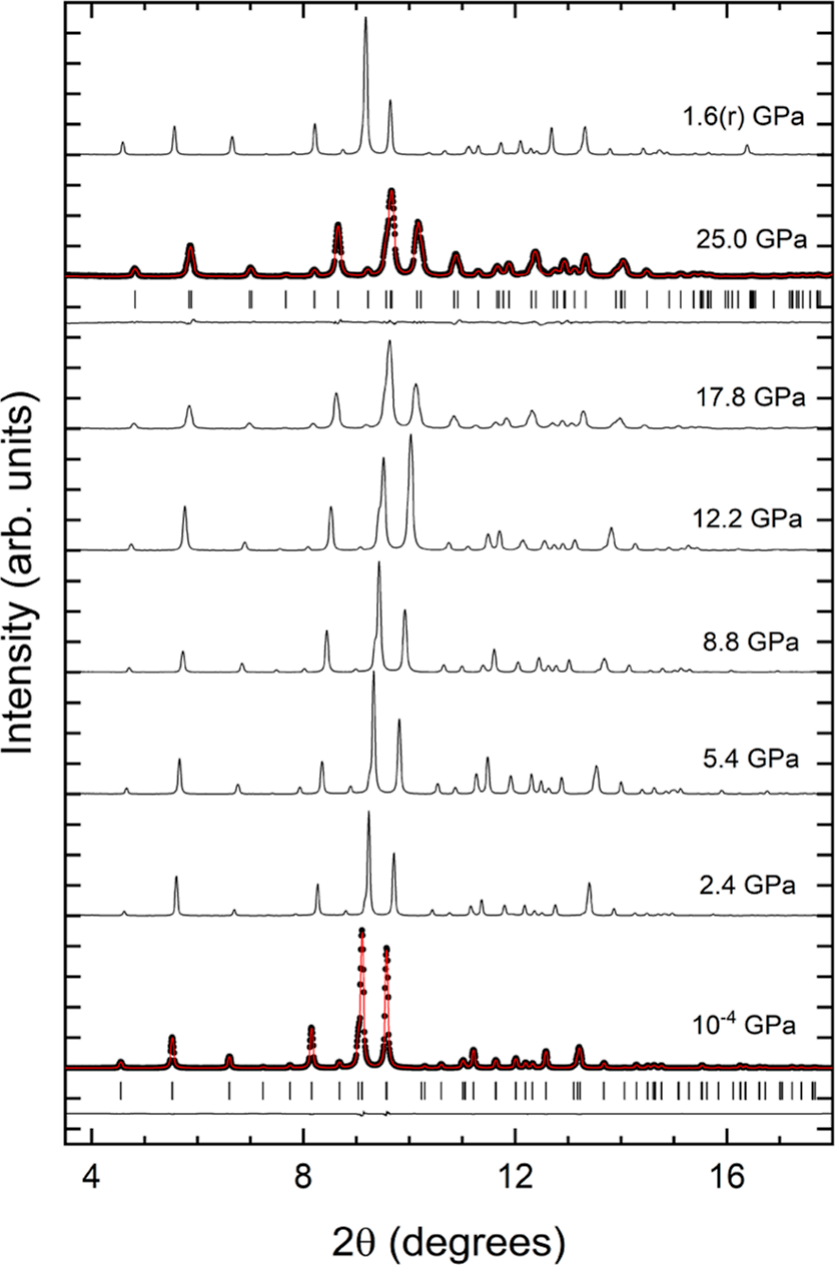
HP XRD patterns measured in KSrPO_4_ (λ = 0.4246 Å). At 10^–4^ and 25 GPa, experiments are shown as solid symbols, refinements as red lines, and residuals as black lines. The ticks show the position of calculated reflections. Pressures are indicated in the figure; (r) is used to identify an experiment performed after decompression.

The structure of KSrPO_4_ is shown in Figure 8. Our study shows that this compound does not undergo any phase transition up to 25 GPa. In Figure 9, we present a selection of XRD patterns collected under compression up to 25 GPa. In this compound, all the XRD patterns can be undoubtedly assigned to the LP phase. This conclusion is supported by the Rietveld refinement of the experiment carried out at 10^–4^ GPa [w*R*_p_ = 3.51%, *R*_p_ = 2.66%, *R*(*F*^2^) = 4.82%, and χ^2^ = 1.60] and the Le Bail fit performed at 25 GPa [w*R*_p_ = 2.97%, *R*_p_ = 2.32%, *R*(*F*^2^) = 4.12%, and χ^2^ = 1.32]; see Figure 9. The above-mentioned ambient-pressure XRD patterns were affected by preferred orientations due to the small number of crystalline domains in the micron-sized X-ray beam. Such effects are the cause of change in the relative intensity of peaks with pressure increasing (see in Figure 9 XRD patterns at 8.8, 12.2, and 17.8 GPa). Above 12.2 GPa, we noticed a peak broadening, which could be caused by non-hydrostatic stresses. However, all peaks in XRD can be undoubtedly identified within the ambient-pressure phase. After the decompression, the XRD pattern measured at the lowest pressure (1.6 GPa) shows that changes in the crystal structure are reversible.

**Figure 10:**
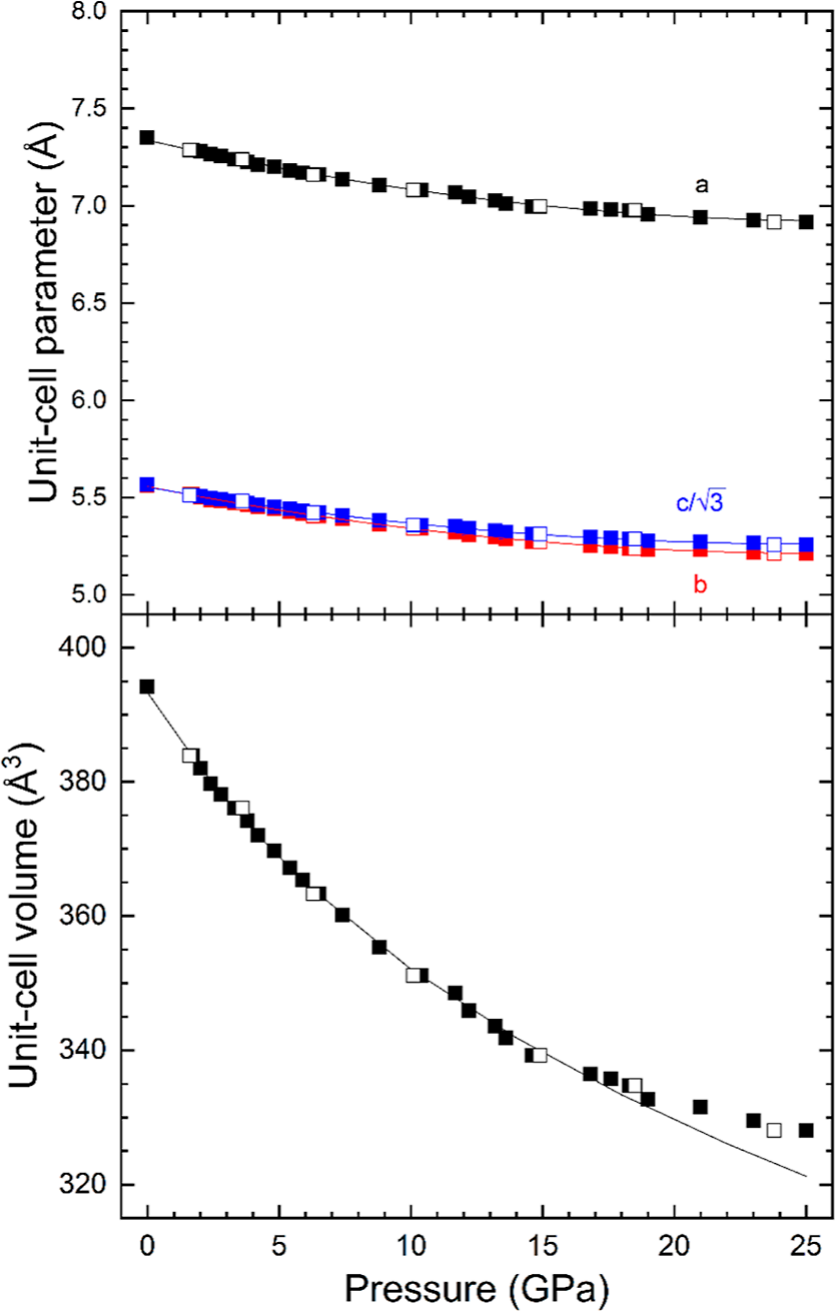
Pressure dependence of unit-cell parameters (top) and volume (bottom). Symbols are results from experiments. Solid (empty) data have been measured during compression (decompression). We plot *c*/√3 to facilitate comparison with *b*. Solid lines are the fits described in the text. Error bars are smaller than symbol size.

From XRD experiments, we have determined the pressure dependence of unit-cell parameters. The results are represented in Figure 10. The unit-cell parameters follow a quadratic dependence on pressure, with a nearly linear behavior up to 10 GPa. Using results from this pressure range, we have determined the linear compressibility for each axis. For KSrPO_4_, we have obtained κ_a_ = 4.24(4) 10^–3^ GPa^–1^, κ_b_ = 4.80(5) 10^–3^ GPa^–1^, and κ_c_ = 4.16(2) 10^–3^ GPa^–1^. The compressibility is thus slightly anisotropic. We have found that the *b*-axis is the most compressible axis. By using the results obtained up to 10 GPa, we have also determined the EOS parameters for KSrPO_4_. Again, we used a third-order Birch–Murnaghan EOS^46^ and the program EosFit7.^47^ The ambient-pressure volume (*V*_0_), ambient-pressure bulk modulus (*K*_0_), its pressure derivative (*K*_0_′), and the implied value of the second pressure derivative (*K*_0_″) are *V*_0_ = 393.9(5) Å^3^, *K*_0_ = 60(2) GPa, *K*_0_′ = 7.2(5), and *K*_0_″ = −0.3195 GPa^–1^, respectively. Both *K*_0_ and *K*_0_′ are larger than those in KCaPO_4_, which indicates that KSrPO_4_ is less compressible than KCaPO_4_. Furthermore, the ratio of axial compressibility of HP-KCaPO_4_ and KSrPO_4_ suggests that the compressibility is strongly reduced in HP-KCaPO_4_ compared to that in KSrPO_4_, which might be due to more compact atomic arrangement. The experimental EOS is shown as a solid line in Figure 10. If the EOS is extrapolated to pressures higher than 10 GPa, it describes very well the results from experiments up to 17.2 GPa. However, beyond this pressure, the experiments show a decrease in the compressibility. We believe that this phenomenon is caused by non-hydrostatic stresses, which usually tends to artificially reduce the compressibility.^48^

### K_2_Ce(PO_4_)_2_ under High Pressure

**Figure 11:**
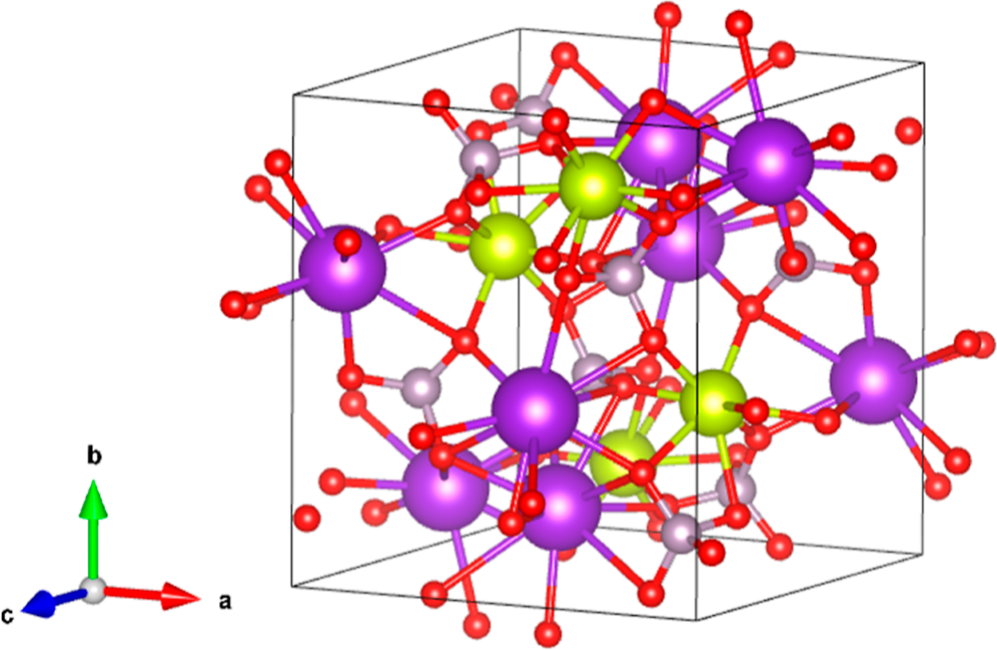
Schematic view of the HP crystal structure of K_2_Ce(PO_4_)_2_. Cerium atoms are represented in yellow, potassium atoms in purple, phosphor atoms in gray, and oxygen atoms in the red color.

**Figure 12:**
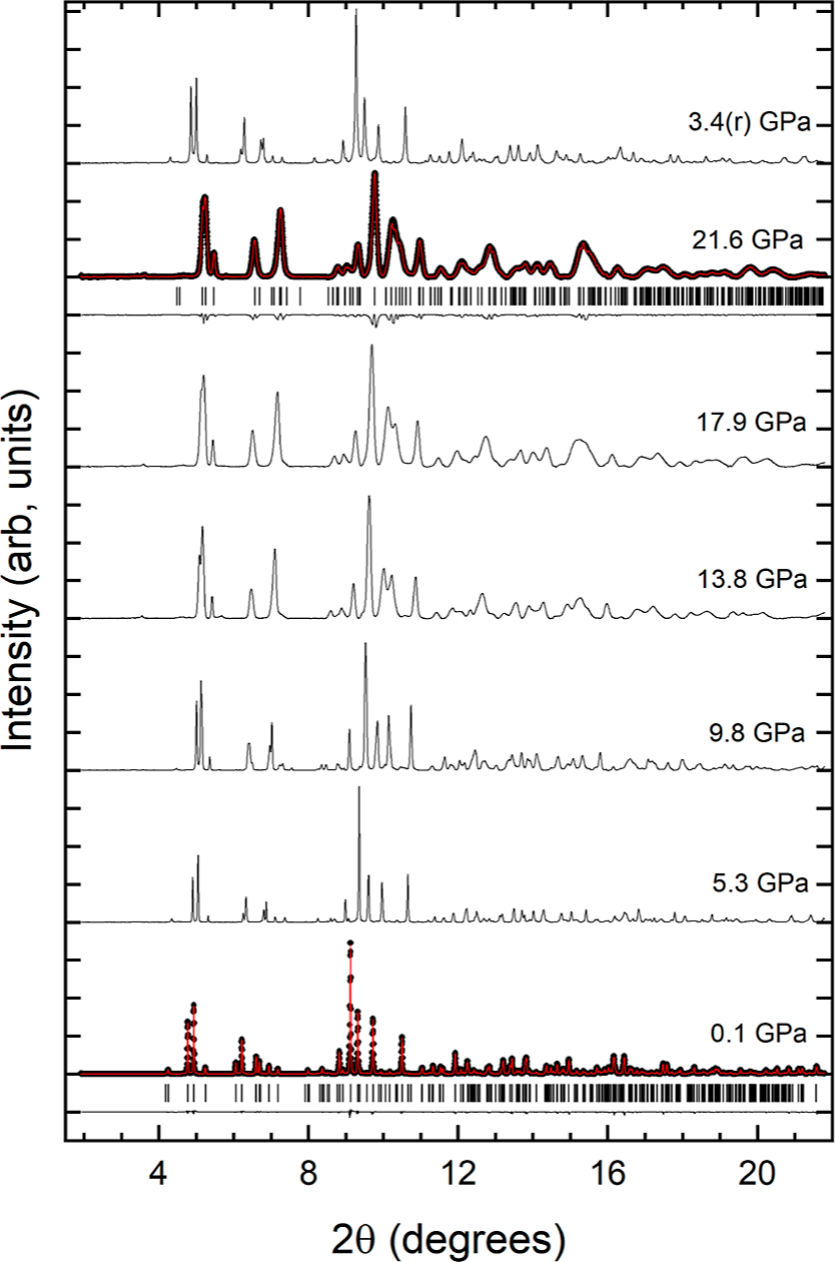
HP XRD patterns measured in K_2_Ce(PO_4_)_2_ (λ = 0.4956 Å). At 0.1 and 21.6 GPa, experiments are shown as solid symbols, refinements as red lines, and residuals as black lines. The ticks show the position of calculated reflections. Pressures are indicated in the figure; (r) is used to identify an experiment performed after decompression.

The structure of K_2_Ce(PO_4_)_2_ obtained from this study is shown in Figure 11. Our experiments show that the ambient-pressure structure (space group *P*2_1_/*n*) remains stable up to 21.6 GPa. In Figure 12, we report a selection of XRD patterns collected under compression. All XRD patterns can be undoubtedly assigned to the LP phase. This conclusion is supported by the Rietveld refinement of the experiment carried out at 0.1 GPa [w*R*_p_ = 3.23%, *R*_p_ = 2.93%, *R*(*F*^2^) = 4.17%, and χ^2^ = 1.49] and the Le Bail fit performed at 21.6 GPa [w*R*_p_ = 4.12%, *R*_p_ = 3.13%, *R*(*F*^2^) = 5.19%, and χ^2^ = 2.03]; see Figure 12. Starting at 13.8 GPa, we observed a peak broadening, which becomes more evident as pressure increases. This may be caused by non-hydrostatic stresses. However, all peaks in XRD can be assigned to the ambient-pressure phase, thus excluding the existence of a phase transition. After the decompression, the XRD pattern at the lowest measured pressure (3.4 GPa) shows that changes in the crystal structure are reversible.

Previous Raman experiments performed under high pressure using 4:1 methanol–ethanol as pressure medium reported a phase transition starting at 8.6 GPa and being completed at 14.4 GPa.^18^ The existence of the phase transition was postulated to explain changes in the intensity of Raman modes and the broadening of internal modes.^20^ One possibility to explain this apparent discrepancy is the presence of different non-hydrostatic conditions in experiments.^49^ However, our pressure medium (16:3:1 methanol–ethanol–water) provides a similar high-pressure environment to 4:1 methanol–ethanol.^50^ A more plausible explanation would be the bridging of the sample between diamonds, which could have strongly influenced the results of high-pressure studies. In our case, we have carefully loaded the diamond-anvil cell to avoid sample bridging. The fact that peak broadening starts in our study at 13.8 GPa but in Raman experiments at 8.6 GPa suggests that sample bridging could have affected the Raman experiments. Such a phenomenon has been found to anticipate phase transitions by more than 10 GPa in oxides like ScVO_4_.^51^ Further studies are needed to understand the influence that deviatoric stresses could have on the HP behavior of K_2_Ce(PO_4_)_2_.

**Figure 13:**
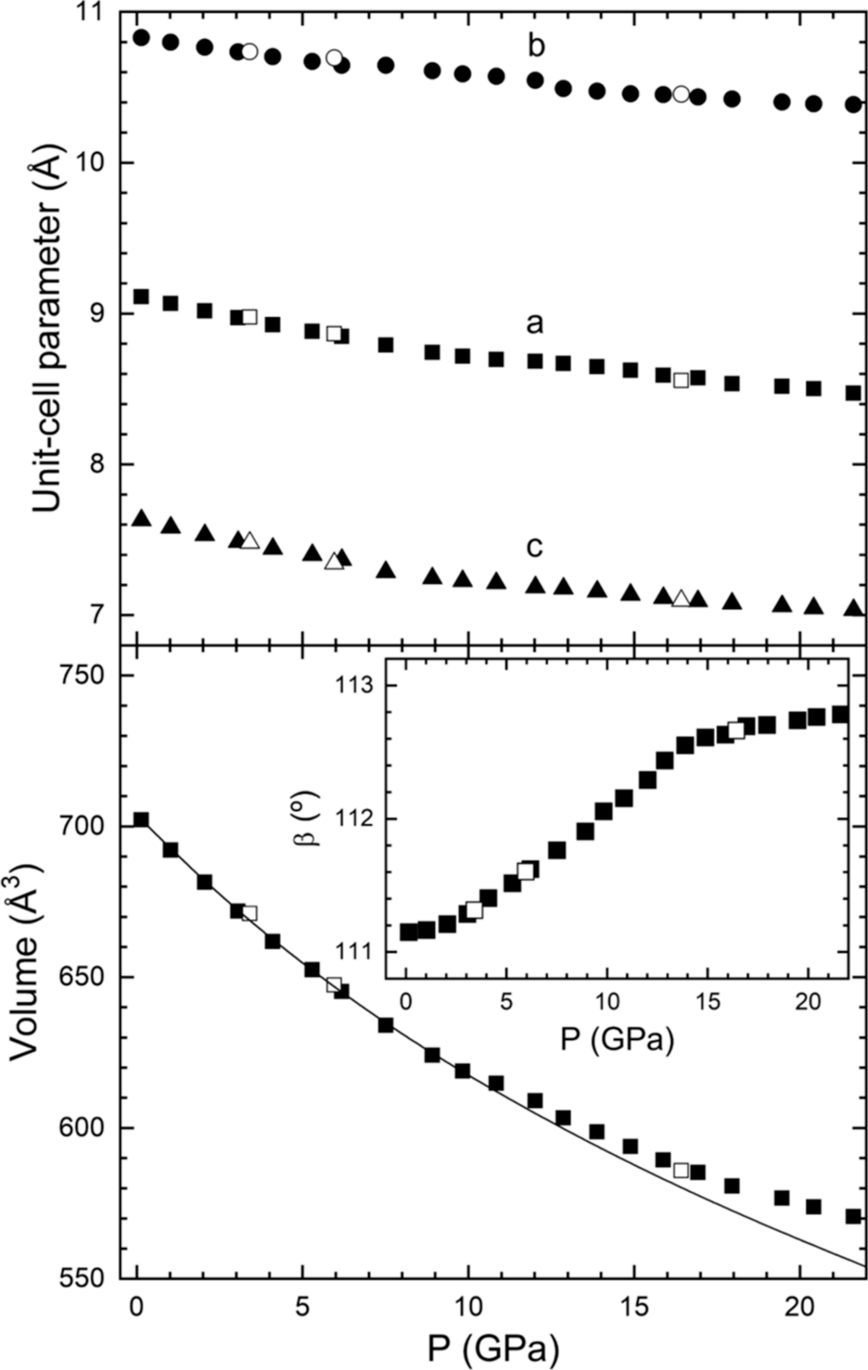
Pressure dependence of unit-cell parameters (top) and volume (bottom). The inset shows the pressure dependence of the angle β. Symbols are results from experiments. Solid (empty) data have been measured during compression (decompression). The solid line is the EOS fit described in the text. Error bars are smaller than symbol size.

From XRD experiments, we have determined the pressure dependence of unit-cell parameters. The results are shown in Figure 13. The lattice parameters *a*, *b*, and *c* follow a similar dependence on pressure, with the last parameter being slightly more compressible than the other. On the other hand, the β angle increases under compression following a non-linear behavior. In the case of a monoclinic structure such as K_2_Ce(PO_4_)_2_, its compressibility should be analyzed by means of the eigenvalues and eigenvectors of the isothermal compressibility tensor.^52^ In our case, we performed this analysis using PASCAL.^53^ We have found that compression is anisotropic and that the maximum, intermediate, and minimum compressibilities are 5.79(9) 10^–3^ GPa^–1^, 4.22(6) 10^–3^ GPa^–1^, and 2.64(2) 10^–3^ GPa^–1^, respectively. The crystallographic directions corresponding to each of the main axes of compressibility are (0.3586, 0, 0.9335), (−0.9903, 0, 0.1388), and (0, 1, 0). By using the results obtained up to 10 GPa, we have determined the EOS parameters for K_2_Ce(PO_4_)_2_. We used a third-order Birch–Murnaghan EOS^46^ and the program EosFit7.^47^ The ambient-pressure volume (*V*_0_), ambient-pressure bulk modulus (*K*_0_), its pressure derivative (*K*_0_′), and the implied value of the second pressure derivative (*K*_0_″) are *V*_0_ = 705.3(1.5) Å^3^, *K*_0_ = 52.0(2.5) GPa, *K*_0_′ = 5.7(6), and *K*_0_″ = −0.1595 GPa^–1^, respectively. The obtained bulk modulus agrees with the value previously obtained from density-functional theory calculations, *K*_0_ = 49 GPa.^18^ The experimental EOS is represented as a solid line in Figure 13. If extrapolated to pressures higher than 10 GPa, it described well the results from experiments up to 13.8 GPa. Beyond this pressure, the experiments show a decrease in the compressibility. As it happens in KSrPO_4_, we consider that this is caused by non-hydrostatic stresses.^31^

## Discussion

We have found that KSrPO_4_ and K_2_Ce(PO_4_)_2_ remain stable up to 26 and 21.6 GPa. In contrast, we have discovered the onset of a phase transition in KCaPO_4_ at 5.6 GPa. The range of stability of the first two compounds is comparable to that of the most stable orthophosphates, for instance, zircon-type,^12^ monazite-type,^13^ olivine-type,^54^ and whitlockite-type.^19,20^ The stability of these compounds is related to the fact that the increasing repulsive and steric stresses induced by pressure can be accommodated by the deformation of the outer shell of the cations and by the tilting of the cationic polyhedra, which are connected by very rigid and uncompressible PO_4_ tetrahedra. The case of KCaPO_4_ is totally different. We are confident that the existence of the phase transition under HP is inherent to the material because the three experiments were performed in the same quasi-hydrostatic environment (same pressure medium). We believe that the transition is favored by two facts: (i) the disorder of the Ca and K atoms at 2d sites, which are randomly distributed in the crystal structure, making it less stable and (ii) the presence of unusually asymmetric Ca(K)O_10_ polyhedra which could also trigger structural instabilities under compression. Notice, in fact, that after the phase transition, the two previously described unusual features of the LP phase of KCaPO_4_ disappear due to the structural reorganization triggered by the transition.

**Figure 14:**
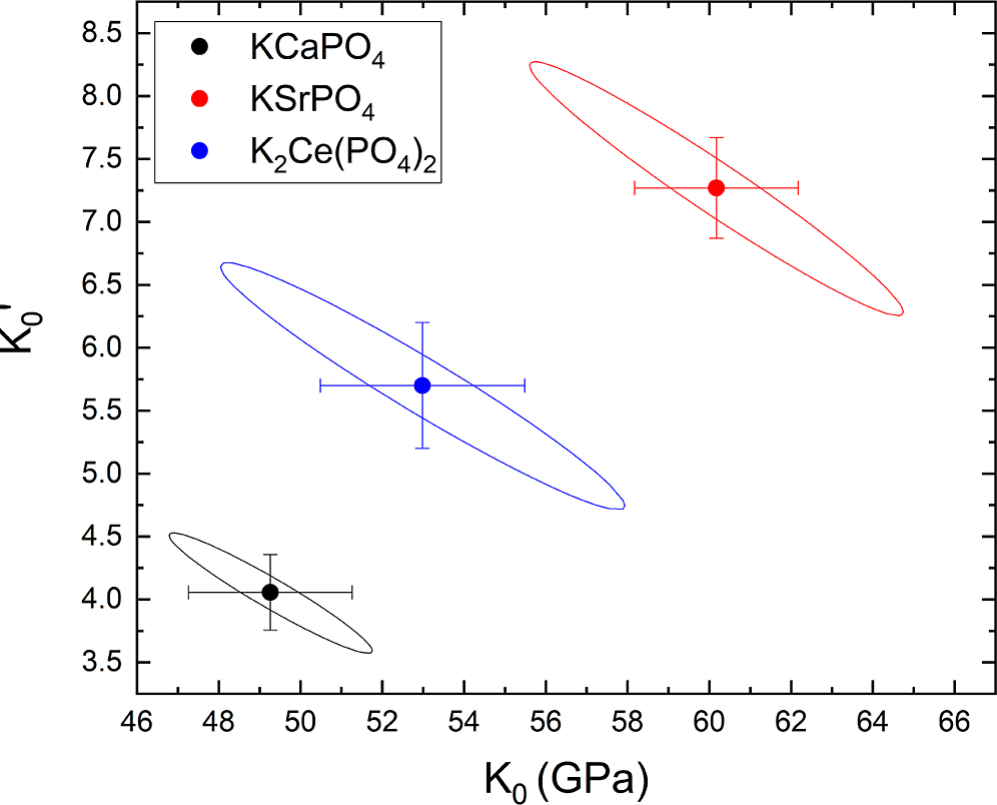
*K*_0_′ versus *K*_0_. Values from the three studied compounds are included (see the legend). Confidence ellipses are shown.

Now, we will compare the bulk modulus of the three studied orthophosphates. Given the fact that *K*_0_ and *K*_0_′ are correlated, a proper comparison can only be made by plotting *K*_0_′ versus *K*_0_, including confidence ellipses of the fits, as reported in Figure 14.^55^ In the figure, it can be seen that there is no overlap between the confidence ellipses and that both *K*_0_ and *K*_0_′ increase following the sequence KCaPO_4_ < K_2_Ce(PO_4_)_2_ < KSrPO_4_. This indicates that their compressibility decreases following the same sequence. On the other hand, the three compounds have bulk moduli comparable to those of the most compressible phosphates in nature. This can be seen in Table 4 where we compare KCaPO_4_, K_2_Ce(PO_4_)_2_, and KSrPO_4_ with previously studied phosphates.^56−70^ Only berlinite-type AlPO_4_^16^ and FePO_4_^15^ and SbPO_4_^67^ are more compressible than the phosphates that we have studied here. Berlinite-type phosphates are more compressible because berlinite is a low-density and open structure which consists of alternating AlO_4_(FeO_4_) and PO_4_ tetrahedra linked with vertices and thus can easily accommodate compression. SbPO_4_ is highly compressible because of the presence of a lone electron pair associated with Sb, which gives the crystal structure a layered characteristic where layers are weakly bonded to each other, favoring a rapid decrease of volume under compression.

**Table 4:** Bulk Moduli of Different Phosphates

compound	*K*_0_ (GPa)	*K*_0_′	reference
KCaPO_4_	49.3(2.0)	4.1(3)	this work
KSrPO_4_	60.2(2.0)	7.3(4)	this work
K_2_Ce(PO_4_)_2_	52.0(2.5)	5.7(6)	this work
K_2_Ce(PO_4_)_2_	49		(18)
FePO_4_	25.4(3)	4	(15)
AlPO_4_	34	4	(14)
α-Ca_3_(PO_4_)_2_	79(2)	4	(56)
β-Ca_3_(PO_4_)_2_	81(3)	4.8(5)	(56)
γ-Ca_3_(PO_4_)_2_	100(13)	5.48(16)	(57)
Sr_3_(PO_4_)_2_	89(2)	6.6(3)	(20)
Pb_3_(PO_4_)_2_	89(4)	5.8(2)	(17)
Ca_9_NaMg(PO_4_)_7_	82(10)	4	(58)
Zn_2_P_2_O_7_	81(2)	5.0(5)	(59)
Ca_5_(PO_4_)_3_0H	97(3)	4	(60)
NaZr_2_(PO_4_)_3_	53		(61)
BaZr(PO_4_)_2_	52	1.2	(16)
Na_3_Fe(PO_4_)(CO_3_)	56(1)	3.3(1)	(62)
Na_3_Mn(PO_4_)(CO_3_)	54(1)	3.3(1)	(62)
Na_3_Mg(PO_4_)(CO_3_)	60(1)	3.9(7)	(63)
LiNiPO_4_	88(2) GPa	3.5(5)	(55)
RbTi_2_(PO_4_)_3_	104		(64)
InPO_4_	97(6)	7(3)	(65)
BiPO_4_ monazite	99(2)	5.8(3)	(66)
BiPO_4_ HT phase	78(4)	4	(66)
CePO_4_ hexagonal	83.9(7)	3.7(3)	(67)
CePO_4_ monazite	120(2)	3.4(4)	(68)
SbPO_4_	36(3)	6(2)	(69)
HoPO_4_	152(3)	4.2(9)	(12)
TmPO_4_	142(1)	5.06(4)	(12)
ScPO_4_	203(7)	4	(13)
LuPO_4_	184(4)–166	4	(13)
YbPO_4_	150(5)	4	(13)
ErPO_4_	168(4)	4	(13)
YPO_4_	132–149(2)–186(5)	4	(13)
GdPO_4_	160(2)	4	(13)
EuPO_4_	159(2)	4	(13)
NdPO_4_	170(2)	4	(13)
LaPO_4_	144(2)	4	(13)
CaTh(PO_4_)_2_	140	4	(70)

As established from the study of different oxides,^10,11^ as a first approximation, the bulk modulus is mainly determined by the compressibility of the most compressible polyhedral units. In the compounds here studied, these are the coordination polyhedra of potassium. The K–O bonds are longer than 3 Å being the weakest bonds in the structures. Harlow^71^ has shown that K–O bond lengths compress significantly with pressure. This conclusion is supported by the fact that K_2_O has a bulk modulus of 39 GPa.^72^ Therefore, the three studied compounds are very compressible compared with most phosphates. This hypothesis is also consistent with the fact that Na_3_Fe(PO_4_)(CO_3_), Na_3_Mn(PO_4_)(CO_3_), and Na_3_Mg(PO_4_)(CO_3_) have bulk moduli in the range of 54–60 GPa as a consequence of the large compressibility of Na–O bonds. Based on these arguments, we can foresee that related compounds like KBaPO_4_ and NaBaPO_4_ would have bulk moduli in the range of 50–60 GPa. It has been proposed that phase-transition pressures of phosphates could be directly correlated to the bulk modulus of the material.^59^ The higher this parameter, the higher the transition pressure. However, this hypothesis is ruled out by the results summarized in Table 4. It is true that the less compressible phosphates (those with the largest bulk modulus), like zircon-type phosphates, are highly stable. Nevertheless, BaZr(PO_4_)_2_, with a bulk modulus of 52 GPa, identical to the bulk modulus of K_2_Ce(PO_4_)_2_, undergoes a phase transition at 0.4 GPa,^9^ while K_2_Ce(PO_4_)_2_ remains stable up to 22 GPa; Pb_3_(PO_4_)_2_ with a bulk modulus of 89 GPa, much larger than the bulk moduli of the phosphates here studied, undergoes a phase transition at 1.7 GPa, while KCaPO_4_, K_2_Ce(PO_4_)_2_, and KSrPO_4_ remain stable up to much higher pressures. Clearly, additional factors, besides compressibility, play a role in crystal stability of phosphates, and further studies will be needed to clarify them. To conclude, we would like to comment that from the conclusions of the present work, it could be foreseen that related materials like KBePO_4_ and KMgPO_4_ would have bulk moduli close to 50 GPa and would retain the LP structure up to similar pressures to KSrPO_4_.

## Conclusions

In summary, we have performed HP powder XRD in KCaPO_4_, K_2_Ce(PO_4_)_2_, and KSrPO_4_. The ambient-pressure crystal structure of KCaPO_4_ has been reassigned, and an HP-induced structural phase transition is observed at 5.5 GPa. The phase transition involves a transformation from the ambient-pressure trigonal structure to an HP orthorhombic structure, which is isostructural to KSrPO_4_. The HP phase remains stable up to 26 GPa, and the phase transition is reversible. No phase transition has been found for K_2_Ce(PO_4_)_2_ and KSrPO_4_ up to 21.6 and 25 GPa, respectively. The pressure–volume data of the different phosphates has been analyzed using the Birch–Murnaghan EOS. The zero-pressure bulk moduli obtained show that these parameters increase following the sequence KCaPO_4_ < K_2_Ce(PO_4_)_2_ < KSrPO_4_. Their linear compressibilities have been also determined. The results show that KCaPO_4_ and KSrPO_4_ are slightly anisotropic and K_2_Ce(PO_4_)_2_ highly anisotropic. The results have been discussed in comparison with the HP behavior of other phosphates. The three studied compounds are among the most compressible phosphates.

## Notes

The data that support the findings of this study are available from the corresponding author upon reasonable request.

## References

[ref1] Achary S. N.; Bevara S.; Tyagi A. K. Recent progress on synthesis and structural aspects of rare-earth phosphates. Coord. Chem. Rev. 2017, 340, 266–297. 10.1016/j.ccr.2017.03.006.

[ref2] Boatner L. A.; Sales B. C.; Monazite Radiation Waste Forms for the Future; Lutze W., Ewing R. C., Eds.; North-Holland: Amsterdam, 1988; pp 495–564.

[ref3] Harper G.; Sommerville R.; Kendrick E.; Driscoll L.; Slater P.; Stolkin R.; Walton A.; Christensen P.; Heidrich O.; Lambert S.; Abbott A.; Ryder K.; Gaines L.; Anderson P. Recycling lithium-ion batteries from electric vehicles. Nature 2019, 575, 75–86. 10.1038/s41586-019-1682-5. 31695206

[ref4] Clavier N.; Dacheux N.; Podor R. Synthesis Characterization, Sintering, and Leaching of β-TUPD/Monazite Radwaste Matrices. Inorg. Chem. 2006, 45, 220–229. 10.1021/ic051607p. 16390059

[ref5] Vergeer P.; Vlugt T. J. H.; Kox M. H. F.; den Hertog M. I.; van der Eerden J. P. J. M.; Meijerink A. Quantum cutting by cooperative energy transfer in Yb_x_Y_1–x_PO_4_:Tb^3+^. Phys. Rev. B: Condens. Matter Mater. Phys. 2005, 71, 014119 10.1103/physrevb.71.014119.

[ref6] Wisniewski D.; Wojtowicz A. J.; Drozdowski W.; Farmer J. M.; Boatner L. A. Rb_3_Lu(PO_4_)_2_:Ce and Cs_3_Lu(PO_4_)_2_:Ce – new promising scintillator materials. Cryst. Res. Techn. 2003, 38, 275–282. 10.1002/crat.200310031.

[ref7] Villa E. M.; Marr C. J.; Jouffret F. J.; Alekseev E. V.; Depmeier W.; Albrecht-Schmitt T. E. Systematic Evolution from Uranyl(VI) Phosphites to Uranium(IV) Phosphates. Inorg. Chem. 2012, 51, 6548–6558. 10.1021/ic3000735. 22646238

[ref8] Lakshminarayana G.; Dao T. D.; Chen K.; Sharma M.; Takeda T.; Brik M. G.; Kityk I. V.; Singh S.; Nagao T. Effect of different surfactants on structural and optical properties of Ce^3+^ and Tb^3+^ co-doped BiPO_4_ nanostructures. Opt. Mater. 2015, 39, 110–117. 10.1016/j.optmat.2014.11.008.

[ref9] Lin C. C.; Xiao Z. R.; Guo G. H.; Chan T. S.; Liu R. S. Versatile Phosphate Phosphors ABPO_4_ in White Light-Emitting Diodes: Collocated Characteristic Analysis and Theoretical Calculations. J. Am. Chem. Soc. 2010, 132, 3020–3028. 10.1021/ja9092456. 20155924

[ref10] Errandonea D.; Manjon F. J. Pressure effects on the structural and electronic properties of ABX_4_ scintillating crystals. Prog. Mater. Science 2008, 53, 711–773.

[ref11] Errandonea D.; Garg A. B. Recent progress on the characterization of the high-pressure behaviour of AVO_4_ orthovanadates. Prog. Mater. Science 2018, 97, 123–169. 10.1016/j.pmatsci.2018.04.004.

[ref12] Gomis O.; Lavina B.; Rodríguez-Hernández P.; Muñoz A.; Errandonea R.; Errandonea D.; Bettinelli M. High-pressure structural, elastic, and thermodynamic properties of zircon-type HoPO_4_ and TmPO_4_. J. Phys.: Condens. Matter 2017, 29, 095401 10.1088/1361-648x/aa516a. 28106012

[ref13] Lacomba-Perales R.; Errandonea D.; Meng Y.; Bettinelli M. High-pressure stability and compressibility of APO_4_ (A = La, Nd, Eu, Gd, Er, and Y) orthophosphates: An x-ray diffraction study using synchrotron radiation. Phys. Rev. B: Condens. Matter Mater. Phys. 2010, 81, 064113 10.1103/physrevb.81.064113.

[ref14] Sharma S. M.; Garg N.; Sikka S. K. High pressure phase transformations in α-AlPO_4_: an x-ray diffraction investigation. J. Phys.: Condens. Matter 2000, 12, 6683–6692. 10.1088/0953-8984/12/30/301.

[ref15] Bull C. L.; Ridley C. J.; Funnell N. P. C. W.; Wilson S. G.; MacLeod S. G. The distortion of two FePO_4_ polymorphs with high pressure. Mater. Adv. 2021, 2, 5096–5104. 10.1039/d1ma00227a.

[ref16] Varma M.; Poswal H. K.; Velaga S. Pressure induced phase transitions in BaZr(PO_4_)_2_ studied using x-ray diffraction, Raman spectroscopy, and first principles calculations. J. Appl. Phys. 2020, 127, 135902 10.1063/1.5144958.

[ref17] Qin F.; Wu X.; Zhai S. M.; Qin S.; Yang K.; Chen D. L.; Li Y. C. Pressure-induced phase transition of lead phosphate Pb_3_(PO_4_)_2_: X-ray diffraction and XANES. Phase Transitions 2014, 87, 1255–1264. 10.1080/01411594.2014.953504.

[ref18] Mishra K. K.; Bevara S.; Ravindran T. R.; Patwe S. J.; Gupta M. K.; Mittal R.; Krishnan R. V.; Achary S. N.; Tyagi A. K. High pressure behavior of complex phosphate K_2_Ce[PO_4_]_2_: Grüneisen parameter and anharmonicity properties. J. Sol. State Chem. 2018, 258, 845–853. 10.1016/j.jssc.2017.12.022.

[ref19] Zhai S.; Wu X. X-ray diffraction study of β-Ca_3_(PO_4_)_2_ at high pressure. Solid State Commun. 2010, 150, 443–445. 10.1016/j.ssc.2009.12.011.

[ref20] Zhai S.; Xue W.; Yamazaki D.; Shan S.; Ito E.; Tomioka N.; Shimojuku A.; Funakoshi K. Compressibility of strontium orthophosphate Sr_3_(PO_4_)_2_ at high pressure. Phys. Chem. Miner. 2011, 38, 357–361. 10.1007/s00269-010-0409-9.

[ref21] Palan C. B.; Koparkar K. A.; Bajaj N. S.; Soni A.; Omanwar S. K. A novel high sensitivity KCaPO_4_:Ce^3+^ phosphor for radiation dosimetry. Res. Chem. Interm. 2016, 42, 7637–7649. 10.1007/s11164-016-2558-z.

[ref22] Tsuta M.; Nakamura S.; Kato A. Micronization of KSrPO_4_:Eu and KBaPO_4_:Eu phosphor particles for white light-emitting diodes by pulsed laser ablation in liquid. Opt. Laser Techn. 2021, 135, 106725 10.1016/j.optlastec.2020.106725.

[ref23] Fang H.; Huang S.; Wei X.; Duan C.; Yin M.; Chen Y. Synthesis and luminescence properties of KCaPO_4_:Eu^2+^,Tb^3+^,Mn^2+^ for white-light-emitting diodes (WLED). J. Rare Earth 2015, 33, 825–829. 10.1016/s1002-0721(14)60491-9.

[ref24] Bredig M. A. Isomorphism and allotropy in compounds of the type A_2_XO_4_. J. Phys. Chem. 1942, 46, 747–764. 10.1021/j150421a009.

[ref25] Launay S.; Mahé P.; Quarton M. Polymorphisme et structure cristalline du monophosphate de sodium et baryum. Mat. Res. Bull. 1992, 27, 1347–1353. 10.1016/0025-5408(92)90100-e.

[ref26] El Ammari L.; El Koumiri M.; Depmeier W.; Hesse K. F.; Elouadi B. The crystal structure of the monophosphate KSrPO_4_. Eur. J. Sol. State and Inorg. Chem. 1997, 34, 563–569.

[ref27] Bevara S.; Achary S. N.; Patwe S. J.; Sinha A. K.; Tyagi A. K. Preparation and crystal structure of K_2_Ce(PO_4_)_2_: a new complex phosphate of Ce(iv) having structure with one-dimensional channels. Dalton Trans. 2016, 45, 980–991. 10.1039/c5dt03288a. 26647831

[ref28] Soni R.; Khan R.; Burange A. S.; Sahani A. J.; Bavera S.; Achary S. N.; Jayaram R. V. Catalytic application of K_2_Ce(PO_4_)_2_ in Knoevenagel condensation - A green protocol. J. Indian Chem. Soc. 2022, 99, 100680 10.1016/j.jics.2022.100680.

[ref29] Bevara S.; Giri P.; Patwe S. J.; Achary S. N.; Mishra R. K.; Kumar A.; Sinha A. K.; Kaushik C. P.; Tyagi A. K. Separation of ^90^Sr from nuclear waste by crystalline complex phosphates of Ce(IV) and Zr(IV). J. Envir. Chem. Engin. 2018, 6, 2248–2261. 10.1016/j.jece.2018.03.013.

[ref30] Bevara S.; Mishra K. K.; Patwe S. J.; Ravindran T. R.; Gupta M. K.; Mittal R.; Krishna P. S. R.; Sinha A. K.; Achary S. N.; Tyagi A. K. Phase Transformation, Vibrational and Electronic Properties of K_2_Ce(PO_4_)_2_: A Combined Experimental and Theoretical Study. Inorg. Chem. 2017, 56, 3335–3348. 10.1021/acs.inorgchem.6b02870. 28263590

[ref31] Bevara S.; Rajeswari B.; Patwe S. J.; Krishna P. S. R.; Shinde A. B.; Achary S. N.; Kadam R. M.; Tyagi A. K. Temperature dependent structural studies and phase transition behavior of K_2_Th(PO_4_)_2_. J. Alloys Compd. 2019, 783, 310–320. 10.1016/j.jallcom.2018.12.315.

[ref32] Gupta S. K.; Rajeshwari B.; Achary S. N.; Tyagi A. K.; Kadam R. M. Controlling the luminescence in K_2_Th(PO_4_)_2_:Eu^3+^ by energy transfer and excitation photon: a multicolor emitting phosphor. New J. Chem. 2020, 44, 14703–14711. 10.1039/d0nj03117h.

[ref33] Fauth F.; Peral I.; Popescu C.; Knapp M. The new Material Science Powder Diffraction beamline at ALBA Synchrotron. Powder Diffr. 2013, 28, S360–S370. 10.1017/s0885715613000900.

[ref34] Lotti P.; Milani S.; Merlini M.; Joseph B.; Alabarse F.; Lausi A. Single-crystal diffraction at the high-pressure Indo-Italian beamline Xpress at Elettra, Trieste. J. Synch. Rad. 2020, 27, 222–229. 10.1107/s1600577519015170. 31868756

[ref35] Dewaele A.; Loubeyre P.; Mezouar M. Equations of state of six metals above 94 GPa. Phys. Rev. B: Condens. Matter Mater. Phys. 2004, 70, 094112 10.1103/physrevb.70.094112.

[ref36] Mao H. K.; Xu J.; Bell P. M. Calibration of the ruby pressure gauge to 800 kbar under quasi-hydrostatic conditions. J. Geophys. Res. 1986, 91, 4673–4676. 10.1029/jb091ib05p04673.

[ref37] Prescher C.; Prakapenka V. B. DIOPTAS: a program for reduction of two-dimensional X-ray diffraction data and data exploration. High Press. Res. 2015, 35, 223–230. 10.1080/08957959.2015.1059835.

[ref38] Rodríguez-Carvajal J. Recent advances in magnetic structure determination by neutron powder diffraction. Phys. B 1993, 192, 55–59.

[ref39] Louer M.; Plevert J.; Louer D. Structure of KCaPO_4_·H_2_O from X-ray powder diffraction data. Acta Cryst. B 1988, 44, 463–467.

[ref40] Boultif A.; Louër D. Powder pattern indexing with the dichotomy method. J. Appl. Cryst. 2004, 37, 724–731. 10.1107/s0021889804014876.

[ref41] Masse R.; Durif A. Chemical preparation and crystal structure refinement of KBaPO_4_ monophosphate. J. Sol. State Chem. 1987, 71, 574–576. 10.1016/0022-4596(87)90270-2.

[ref42] Bastide J. P. Systématique simplifiée des composés ABX_4_ (X = O^2–^, F^–^) et evolution possible de leurs structures cristallines sous pression. J. Sol. State Chem. 1987, 71, 115–120. 10.1016/0022-4596(87)90149-6.

[ref43] Errandonea D. High-pressure phase transitions and properties of MTO_4_ compounds with the monazite-type structure. Phys. Stat. Sol. B 2017, 254, 1700016 10.1002/pssb.201700016.

[ref44] Ojima K.; Nishihata Y.; Sawada D. A. Structure of potassium sulfate at temperatures from 296 K down to 15 K. Acta Cryst. B 1995, 51, 287–293. 10.1107/s0108768194013327.

[ref45] McGinnety J. A. Redetermination of the structures of potassium sulphate and potassium chromate: the effect of electrostatic crystal forces upon observed bond lengths. Acta Cryst. B 1972, 28, 2845–2852. 10.1107/s0567740872007022.

[ref46] Birch F. Finite Elastic Strain of Cubic Crystals. Phys. Rev. 1947, 71, 809–824. 10.1103/physrev.71.809.

[ref47] Gonzalez-Platas J.; Alvaro M.; Nestola F.; Angel R. EosFit7-GUI: a new graphical user interface for equation of state calculations, analyses and teaching. J. Appl. Cryst. 2016, 49, 1377–1382. 10.1107/s1600576716008050.

[ref48] Errandonea D.; Muñoz A.; Gonzalez-Platas J. Comment on “High-pressure x-ray diffraction study of YBO_3_/Eu^3+^, GdBO_3_, and EuBO_3_: Pressure-induced amorphization in GdBO_3”_ [J. Appl. Phys. 115, 043507 (2014)]. J. Appl. Phys. 2014, 115, 216101 10.1063/1.4881057.

[ref49] Mall A. K.; Garg N.; Verma A. K.; Errandonea D.; Chitnis A. V.; Srihari V.; Gupta R. Discovery of high-pressure post-perovskite phase in HoCrO_3_. J. Phys. Chem. Sol. 2023, 172, 111078 10.1016/j.jpcs.2022.111078.

[ref50] Klotz S.; Chervin J. C.; Munsch P.; Le Marchand G. Hydrostatic limits of 11 pressure transmitting media. J. Phys. D: Appl. Phys. 2009, 42, 075413 10.1088/0022-3727/42/7/075413.

[ref51] Garg A. B.; Errandonea D.; Rodríguez-Hernández P.; Muñoz A. ScVO_4_ under non-hydrostatic compression: a new metastable polymorph. J. Phys.: Condens. Matter 2017, 29, 055401 10.1088/1361-648x/29/5/055401. 27941236

[ref52] Knight K. S. Analytical expressions to determine the isothermal compressibility tensor and the isobaric thermal expansion tensor for monoclinic crystals: Application to determine the direction of maximum compressibility in jadeite. Phys. Chem. Miner. 2010, 37, 529–533. 10.1007/s00269-009-0353-8.

[ref53] Cliffe M. J.; Goodwin A. L. PASCal a principal axis strain calculator for thermal expansion and compressibility determination. J. Appl. Cryst. 2012, 45, 1321–1329. 10.1107/s0021889812043026.

[ref54] Bandiello E.; Errandonea D.; Pellicer-Porres J.; Garg A. B.; Rodriguez-Hernandez P.; Muñoz A.; Martinez-Garcia D.; Rao R.; Popescu C. Effect of High Pressure on the Crystal Structure and Vibrational Properties of Olivine-Type LiNiPO_4_. Inorg. Chem. 2018, 57, 10265–10276. 10.1021/acs.inorgchem.8b01495. 30052035

[ref55] Anzellini S.; Burakovsky L.; Turnbull R.; Bandiello E.; Errandonea D. P-V-T Equation of State of Iridium Up to 80 GPa and 3100 K. Crystals 2021, 11, 452 10.3390/cryst11040452.

[ref56] Scott P. R.; Crow J. A.; LeGeros R. Z.; Kruger M. B. A pressure-induced amorphous phase transition in magnesium-substituted-tricalcium phosphate. Sol. State Comm. 2011, 151, 1609–1611. 10.1016/j.ssc.2011.07.019.

[ref57] Zhai S.; Liu X.; Shieh S. R.; Zhang L.; Ito E. Equation of State of Tricalcium Phosphate, Ca_3_(PO_4_)_2_, to Lower Mantle Pressures. Am. Miner. 2009, 94, 1388–1391. 10.2138/am.2009.3160.

[ref58] Jia M.; Hu X.; Liu Y.; Jiang S.; Wu X.; Zhai S. X-ray diffraction and Raman spectra of merrillite at high pressures. High Pres. Res 2020, 40, 411–422. 10.1080/08957959.2020.1798945.

[ref59] Shakhvorostov D.; Müser M. H.; Mosey N. J.; Song Y.; Norton P. R. Correlating cation coordination, stiffness, phase transition pressures, and smart materials behavior in metal phosphates. Phys. Rev. B: Condens. Matter Mater. Phys. 2009, 79, 094107 10.1103/physrevb.79.094107.

[ref60] Brunet F.; Allan D. R.; Redfern S. A. T.; Angel R. J.; Miletich R.; Reichmann H. J.; Sergent J.; Hanfland M. Compressibility and thermal expansivity of synthetic apatites, Ca_5_(PO_4_)_3_X with X = OH, F and Cl. Eur. J. Miner. 1999, 11, 1023–1036. 10.1127/ejm/11/6/1023.

[ref61] Kamali K.; Ravindran T. R.; Chandra Shekar N. V.; Pandey K. K.; Sharma S. M. Pressure induced phase transformations in NaZr_2_(PO_4_)_3_ studied by X-ray diffraction and Raman spectroscopy. J. Sol. State Chem. 2015, 221, 285–290. 10.1016/j.jssc.2014.10.017.

[ref62] Gao J.; Huang W.; Wu X.; Qin S. High pressure experimental studies on Na_3_Fe(PO_4_)(CO_3_) and Na_3_Mn(PO_4_)(CO_3_): Extensive pressure behaviors of carbonophosphates family. J. Phys. Chem. Solids 2018, 115, 248–253. 10.1016/j.jpcs.2017.12.046.

[ref63] Gao J.; Huang W. F.; Wu X.; Fan D. W.; Wu Z. Y.; Xia D. G.; Qin S. Compressibility of carbonophosphate bradleyite Na_3_Mg(CO_3_)(PO_4_) by X-ray diffraction and Raman spectroscopy. Phys. Chem. Miner. 2015, 42, 191–201. 10.1007/s00269-014-0710-0.

[ref64] Clavier N.; Wallez G.; Dacheux H.; Bregiroux D.; Quarton M.; Beaunier P. Synthesis Raman and Rietveld analysis of thorium diphosphate. J. Sol. State Chem. 2008, 181, 3352–3356. 10.1016/j.jssc.2008.09.013.

[ref65] Dwivedi A.; Kaiwart R.; Varma M.; Velaga S.; Poswal H. K. High-pressure structural investigations on InPO_4_. J. Sol. State Chem. 2020, 282, 121065 10.1016/j.jssc.2019.121065.

[ref66] Errandonea D.; Gomis O.; Santamaría-Perez D.; García-Domene B.; Muñoz A.; Rodríguez-Hernández P.; Achary S. N.; Tyagi A. K.; Popescu C. Exploring the high-pressure behavior of the three known polymorphs of BiPO_4_: Discovery of a new polymorph. J. Appl. Phys. 2015, 117, 105902 10.1063/1.4914407.

[ref67] Bandiello E.; Errandonea D.; Ferrari S.; Pellicer-Porres J.; Martínez-García D.; Achary S. N.; Tyagi A. K.; Popescu C. Pressure-Induced Hexagonal to Monoclinic Phase Transition of Partially Hydrated CePO_4_. Inorg. Chem. 2019, 58, 4480–4490. 10.1021/acs.inorgchem.8b03648. 30864787

[ref68] Huang T.; Lee J. S.; Kung J.; Lin C. M. Study of monazite under high pressure. Solid State Commun. 2010, 150, 1845–1850. 10.1016/j.ssc.2010.06.042.

[ref69] Pereira A. L. J.; Santamaría-Pérez D.; Vilaplana R.; Errandonea D.; Popescu C.; da Silva E. L.; Sans J. A.; Rodríguez-Carvajal J.; Muñoz A.; Rodríguez-Hernández P.; Mujica A.; Radescu S. E.; Beltrán A.; Otero-de-la-Roza A.; Nalin M.; Mollar M.; Manjón F. J. Experimental and Theoretical Study of SbPO_4_ under Compression. Inorg. Chem. 2020, 59, 287–307. 10.1021/acs.inorgchem.9b02268. 31876414

[ref70] Raison P. E.; Heathman S.; Wallez G.; Zvoriste C. E.; Bykov D.; Ménard G.; Suard E.; Popa K.; Dacheux N.; Konings R. J. M.; Caciuffo R. Structure and nuclear density distribution in the cheralite—CaTh(PO_4_)_2_: studies of its behaviour under high pressure (36 GPa). Phys. Chem. Miner. 2012, 39, 685–692. 10.1007/s00269-012-0522-z.

[ref71] Harlow G. E. Structure refinement of a natural K-rich diopside: The effect of K on the average structure. Am. Miner. 1996, 81, 632–638. 10.2138/am-1996-5-610.

[ref72] Moakafi M.; Khenata R.; Bouhemadou A.; Khachai H.; Amrani B.; Rached D.; Rérat M. Electronic and optical properties under pressure effect of alkali metal oxides. Eur. Phys. J. B 2008, 64, 35–42. 10.1140/epjb/e2008-00286-6.

